# Spatio‐temporal changes in pre‐exposure prophylaxis uptake among MSM in mainland France between 2016 and 2021: a Bayesian small area approach with MSM population estimation

**DOI:** 10.1002/jia2.26089

**Published:** 2023-05-23

**Authors:** Haoyi Wang, Jean‐Michel Molina, Rosemary Dray‐Spira, Axel J. Schmidt, Ford Hickson, David van de Vijver, Kai J. Jonas

**Affiliations:** ^1^ Department of Work and Social Psychology Maastricht University Maastricht the Netherlands; ^2^ Viroscience Department Erasmus Medical Centre Rotterdam the Netherlands; ^3^ Department of Infectious Diseases Hôpital Saint‐Louis University of Paris Cité Paris France; ^4^ EPI‐PHARE, French National Agency for Medicines and Health Products Safety (ANSM) and French National Health Insurance (CNAM) Saint‐Denis France; ^5^ Sigma Research, London School of Hygiene and Tropical Medicine London UK

**Keywords:** Bayesian spatio‐temporal analysis, HIV prevention, men who have sex with men (MSM), MSM population estimation, pre‐exposure prophylaxis (PrEP), small area estimation

## Abstract

**Introduction:**

In France, oral pre‐exposure prophylaxis (PrEP) for HIV prevention has been publicly available since 2016, mainly targeting at men who have sex with men (MSM). Reliable and robust estimations of the actual PrEP uptake among MSM on a localized level can provide additional insights to identify and better reach marginalized MSM within current HIV prevention service provision. This study used national pharmaco‐epidemiology surveillance data and regional MSM population estimations to model the spatio‐temporal distribution of PrEP uptake among MSM in France 2016–2021 to identify marginalized MSM at risk for HIV and increase their PrEP uptake.

**Methods:**

We first applied Bayesian spatial analyses with survey‐surveillance‐based HIV incidence data as a spatial proxy to estimate the size of (1) regional HIV‐negative MSM populations and (2) MSM who could be eligible for PrEP use according to French PrEP guidelines. We then applied Bayesian spatio‐temporal ecological regression modelling to estimate the regional prevalence and relative probability of the overall‐ and new‐PrEP uptake from 2016 to 2021 across France.

**Results:**

HIV‐negative and PrEP‐eligible MSM populations vary regionally across France. *Île‐de‐France* was estimated to have the highest MSM density compared to other French regions. According to the final spatio‐temporal model, the relative probability of overall PrEP uptake was heterogeneous across France but remained stable over time. Urban areas have higher‐than‐average probabilities of PrEP uptake. The prevalence of PrEP use increased steadily (ranging from 8.8% [95% credible interval 8.5%;9.0%] in Nouvelle‐Aquitaine to 38.2% [36.5%;39.9%] in Centre‐Val‐de‐Loire in 2021).

**Conclusions:**

Our results show that using Bayesian spatial analysis as a novel methodology to estimate the localized HIV‐negative MSM population is feasible and applicable. Spatio‐temporal models showed that despite the increasing prevalence of PrEP use in all regions, geographical disparities and inequalities of PrEP uptake continued to exist over time. We identified regions that would benefit from greater tailoring and delivery efforts. Based on our findings, public health policies and HIV prevention strategies could be adjusted to better combat HIV infections and to accelerate ending the HIV epidemic.

## 1 INTRODUCTION

Ending the HIV epidemic by 2030 [1] requires HIV prevention services to better reach underserved and marginalized populations [2]. HIV oral pre‐exposure prophylaxis (PrEP) is highly effective [3, 4, 5, 6, 7]. In France, it has been fully covered by the national PrEP delivery programme since 2016 [8] and annual absolute numbers of PrEP delivery per administrative region are publicly available [9]. The uptake has not been as high as some authorities expected [8]. By examining geographic disparities and barriers to PrEP use [10], marginalized populations may be identified, and HIV prevention could be improved by geographic area, instead of treating the country homogenously.

One challenge to such tailoring is the unknown size of the regional HIV‐negative men who have sex with men (MSM) populations. Studies exploring methods to estimate MSM population sizes of France [11, 12, 13] have remained on the national level. Information on a smaller geographical scale, such as administrative regional level, remains unavailable and does not allow matching with the regional PrEP delivery data. For concise PrEP uptake surveillance, the size of (1) regional HIV‐negative MSM populations and (2) those eligible according to French PrEP guidelines (hereafter eligible MSM population) should be estimated at a regional level. Furthermore, it is important to understand both psychosocial and behavioural facilitators and barriers to PrEP uptake and spatio‐temporal trends to unravel co‐variation [14]. These psychosocial and behavioural determinants can differ regionally, too. Such joint modelling has shown to improve the robustness of estimates [14, 15].

To close this gap, we first aimed to estimate the size of regional HIV‐negative MSM populations, as well as the number of PrEP‐eligible MSM. In addition, we sought to investigate how PrEP uptake among eligible MSM differed across regions over time since its public introduction in France in 2016.

## 2 METHODS

### 2.1 Study population and data sources

For the modelling analysis, we included 12 metropolitan regions which are in mainland France (hereafter France) and excluded the metropolitan region of *Corse* and the five French overseas regions (*DOM‐TOM*).

To conduct our analysis, we drew on data from three different sources. We first retrieved 2017 self‐reported data on newly diagnosed HIV among MSM from the French subsample of the European‐MSM‐Internet‐Survey (EMIS‐2017, www.emis2017.eu). EMIS‐2017 recruited 10,385 MSM living in mainland France between 19 October 2017 and 30 January 2018 [16]. Ethical approval and informed consent for EMIS‐2017 was obtained from the Observational Research Ethics Committee at the London School of Hygiene & Tropical Medicine (review reference 14421/RR/8805). We also retrieved 2017 notification data on newly diagnosed HIV among MSM surveillance unadjusted data at the French metropolitan regional level from *Santé Publique France* [17]. Lastly, pharmaco‐epidemiology surveillance data of PrEP delivery for the years 2016–21 were derived from *épidémiologie des produits de santé* (EPI‐PHARE) [9]. All three data sources were broken down to regional levels, using the 12 regions described above. For more information on the source data and their functions in this study, see Supplement S1.

### 2.2 Bayesian spatio‐temporal analysis

Bayesian spatio‐temporal analysis is a well‐established method for small‐area‐estimations [15, 18, 19, 20, 21, 22]. Bayesian spatio‐temporal analysis can account for a number of sources of error or bias, including spatial autocorrelation between neighbouring regions and proximity (Supplement S2); time‐dependent autocorrelation between consecutive time periods; and uncertainties due to instability of estimates in sparsely populated areas [18, 20, 21]. Following previous approaches [10, 15], we used the Integrated Nested Laplace Approximation (INLA) and appointed a Penalized Complexity prior for the precision of the exchangeable random effects [23]. In Bayesian statistics, prior is the probability of an event before data are added to the analysis. This assesses the probability of an outcome based on current knowledge. Prior probability can be compared with posterior probability. We then employed the re‐parameterized Besag–York–Mollie (BYM2) model [23], which specifies the spatially structured residual using an intrinsic conditional autoregressive distribution [24].

We first applied this method as a novel approach to estimate the HIV‐negative MSM and the PrEP‐eligible MSM population, both at the regional level. Bayesian spatial analysis was applied to estimate the regional posterior relative risk (RR) of newly diagnosed HIV among MSM in 2017 using self‐reported data from EMIS‐2017. Given the robust estimations using survey data by the Bayesian spatial analysis [15], we assumed the posterior RRs are comparable to the true RR. We then calculated the nationwide HIV incidence in 2017, using the newly diagnosed HIV surveillance unadjusted data among MSM from *Santé Publique France* as a numerator, and the estimated total HIV‐negative MSM population in France by Ndawinz et al. [25] as a denominator.

Using the estimated nationwide HIV incidence, while knowing the regional posterior RRs of newly diagnosed HIV among MSM, regional HIV incidences were calculated. Lastly, with unadjusted surveillance data of newly diagnosed HIV among MSM in 2017, and accounting to the French PrEP eligibility criteria [26] using EMIS‐17 self‐reported data for the regional PrEP‐eligible proportions, we estimated the regional HIV‐negative and PrEP‐eligible MSM population. For a more detailed methods description, see Supplement S3.

**Figure 1:**
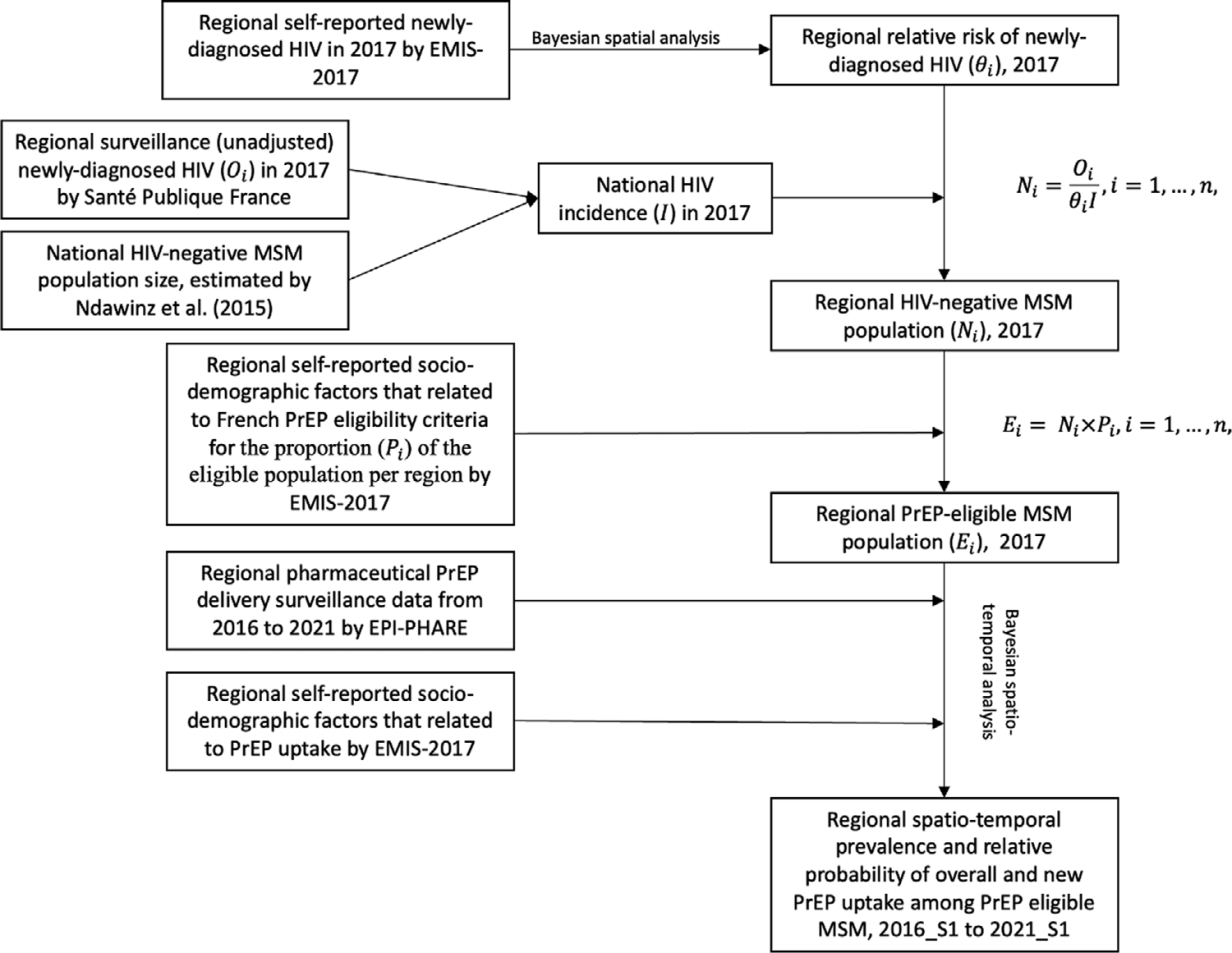
Simplified modelling process schema. Note: For a more detailed methods description, see Supplements S3 and S4.

Next, using the pharmaco‐epidemiology PrEP surveillance data, we applied this method to describe the posterior prevalence and relative probability of overall PrEP uptake among MSM by region and period from 2016 to 2021, and the additional new PrEP uptake among MSM (results in Supplements S9–S13). Figure 1 summarizes a simplified modelling process showing how source data are involved.

### 2.3 Spatio‐temporal modelling of PrEP uptake

#### 2.3.1 Spatio‐temporal modelling analysis (null model)

We first modelled the posterior prevalence and relative probability of overall and new PrEP uptake among PrEP‐eligible MSM with a space–time interaction (null model). We split every year into two periods (S1 [January–June] and S2 [July–December]), leading to 11 periods in total (2016_S1‐2021_S1). For detailed model assumptions and parameter appointments, see Supplement S4.

#### 2.3.2 Ecological regression modelling analysis (univariable models and multivariable final model)

Additional to the null model, we hypothesized that PrEP uptake among MSM across France can be influenced by psychosocial and behavioural determinants [14]. These determinants were taken from EMIS‐2017 and included: The proportion of MSM intending to use PrEP (defined as being quite‐ and very‐likely to use PrEP when PrEP is available and affordable), PrEP knowledge (defined as answering all three questions on PrEP knowledge correctly), being diagnosed with syphilis, gonorrhoea or chlamydia in the preceding 5 years (for this determinant, we only made use of data from the non‐French speaking respondents, given a translation issue in the French‐language version of EMIS‐2017 [27] (see Limitations section), engaging in condomless anal intercourse (CAI) with non‐steady male partners, reporting male non‐steady CAI partners, combining substances with sex (“chemsex”) in the previous 12 months, chemsex with multiple partners in the preceding 12 months, having steady or non‐steady partners with diagnosed HIV and demographic determinants, including low education (defined as less than 3 years of education beyond 16 years of age), unemployment and financial struggling. Descriptions of all variables can be found in the method paper of EMIS‐2017 [16].

We applied a spatio‐temporal ecological regression modelling technique [15, 18] which takes these determinants of PrEP uptake into account. We assumed that the estimated regional determinants among HIV‐negative MSM, summarized by EMIS‐2017 data, are comparable to the general MSM population and stable over the study periods. We first conducted univariable models that only included one of the selected regional determinants. We then conducted multivariable models with those determinants indicated significant by the univariable models, to evaluate the explained variance of PrEP uptake in France. We selected the final model using the backward approach by comparing the deviance information criterion (DIC) of the models, in which the smaller the value, the better the goodness‐of‐fit [28, 29]. For detailed assumptions and parameter appointments of the spatio‐temporal regression models, see Supplement S4.

All analyses were conducted in R (version 4.2.1). For all Bayesian modelling analyses with INLA, we used the R‐INLA package (version 21.05.02) [18].

## 3 RESULTS

### 3.1 Study population characteristics and eligible MSM population estimations

**Figure 2:**
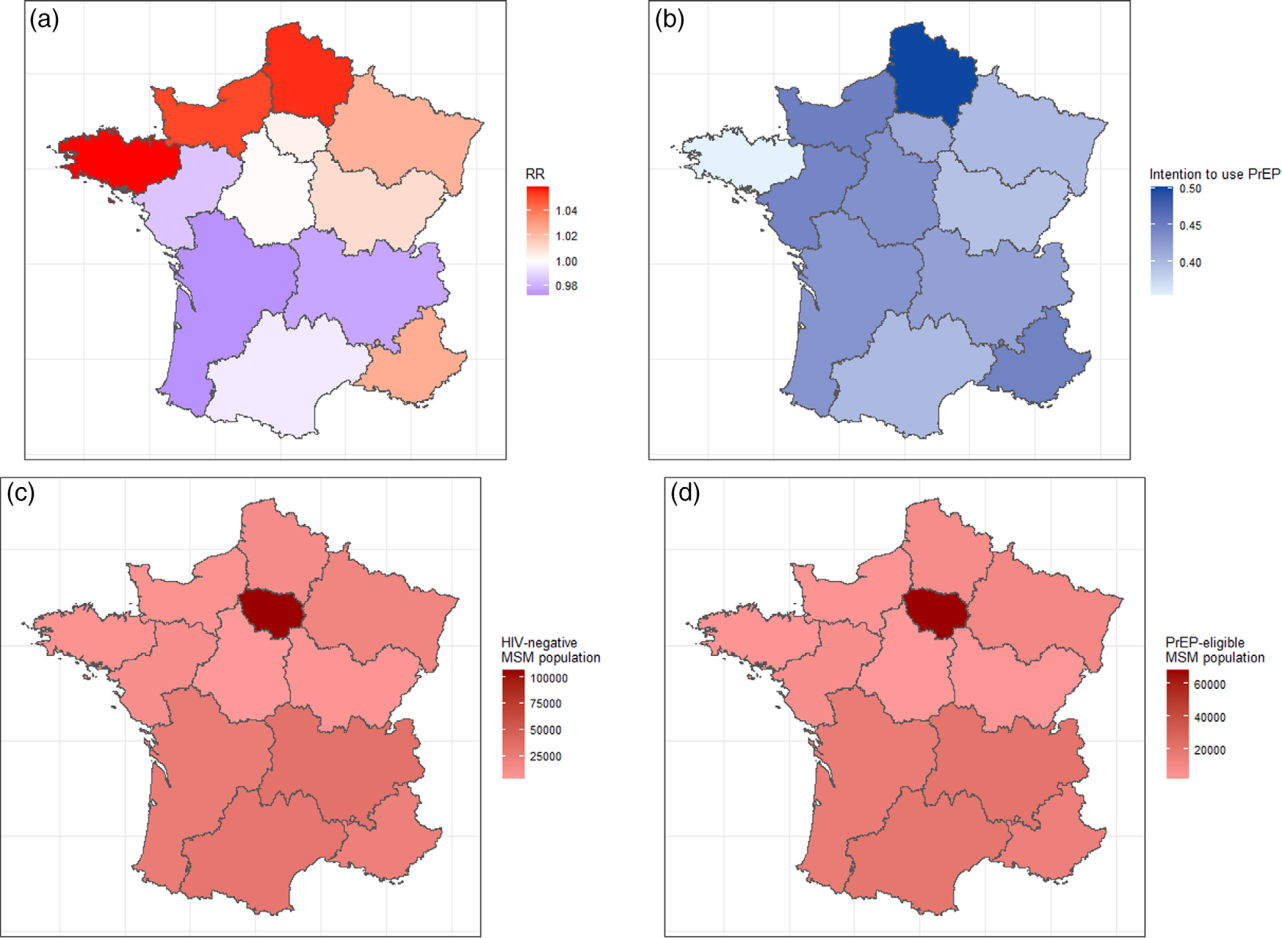
Choropleth map of the (a) projected HIV incidence relative risk; (b) intention to use PrEP among MSM; (c) estimated HIV‐negative MSM population; (d) estimated PrEP‐eligible MSM population, by regions in mainland France, 2017.

A total of 1147 individuals newly diagnosed with HIV were reported by *Santé Publique France* (unadjusted) for the different regions in 2017 (Table 1) [17]. The regional posterior RR ranged from 0.972 (95% credible interval [95% CrI] 0.66;1.28) in Nouvelle‐Aquitaine to 1.055 (95% CrI 0.761;1.27) in Hauts‐de‐France (Table 1 and Figure 2a). The HIV‐negative MSM population sizes across France were estimated from 2746 (95% CrI 2.031;3.940) in Centre‐Val‐de‐Loire to 106,521 (95% CrI 84.040;139.553) in Île‐de‐France (Table 1 and Figure 2c). Similarly, the PrEP‐eligible MSM population ranged from 1834 (95% CrI 1.357;2.632) in Centre‐Val‐de‐Loire to 68,073 (95% CrI 53.706;89.182) in Île‐de‐France (Table 1 and Figure 2d).

**Table 1:** Modelled relative risk of HIV incidence, estimated MSM at risk for HIV, MSM who meet French PrEP guidelines, per region in mainland France, 2017

Region	Number newly diagnosed HIV [17]^a^	Projected RR 2017	(95% CrI)	Estimated number of HIV‐negative MSM	(95% CrI)	Estimated number of PrEP‐eligible MSM	(95% CrI)	Men aged 20–75 years in 2017 [30]^b^	(% HIV‐negative MSM)	Proportion intending to use PrEP^c^
Auvergne‐Rhône‐Alpes	132	0.977	(0.694;1.266)	33,759	(26,048;47,504)	20,494	(15,813;28,838)	2,585,405	(1.3%)	0.42
Bourgogne‐Franche‐Comté	24	1.011	(0.708;1.388)	5934	(4322;8475)	3240	(2360;4628)	918,896	(0.6%)	0.39
Bretagne	32	1.059	(0.738;1.603)	7551	(4991;10,835)	4047	(2675;5807)	1,078,951	(0.7%)	0.35
Centre‐Val de Loire	11	1.001	(0.698;1.354)	2746	(2031;3940)	1834	(1357;2632)	828,340	(0.3%)	0.43
Grand Est	74	1.024	(0.737;1.4)	18,065	(13,214;25,098)	10,566	(7729;14,679)	1,844,353	(0.9%)	0.40
Hauts‐de‐France	56	1.055	(0.761;1.527)	13,260	(9164;18,403)	8065	(5574;11,194)	1,936,388	(0.7%)	0.50
Île‐de‐France	428	1.004	(0.767;1.273)	106,521	(84,040;139,553)	68,073	(53,706;89,182)	3,961,964	(2.7%)	0.41
Normandie	32	1.051	(0.758;1.524)	7606	(5249;10,553)	4532	(3127;6288)	1,074,068	(0.7%)	0.45
Nouvelle‐Aquitaine	100	0.972	(0.66;1.28)	25,707	(19,520;37,863)	16,521	(12,545;24,333)	1,936,388	(1.3%)	0.43
Occitanie	121	0.995	(0.699;1.328)	30,399	(22,765;43,272)	19,177	(14,361;27,298)	1,898,052	(1.6%)	0.40
Pays de la Loire	43	0.985	(0.661;1.325)	10,907	(8111;16,268)	6911	(5139;10,307)	1,205,955	(0.9%)	0.44
Provence‐Alpes‐Côte d'Azur	94	1.025	(0.727;1.425)	22,921	(16,481;32,322)	14,469	(10,403;20,403)	1,607,019	(1.4%)	0.45

^a^ Data retrieved from French surveillance unadjusted data.

^b^ Data retrieved from Institut national de la statistique et des études économiques.

^c^ Data retrieved from EMIS‐2017.

We also observed a heterogenous distribution of the estimated determinants of PrEP uptake among MSM by EMIS‐2017, on a regional level. For example, the proportion intending to use PrEP ranged from 35% in Bretagne to 50% in Hauts‐de‐France (Table 1 and Figure 2b). For details, see Supplement S5.

### 3.2 Prevalence and relative probability of the overall PrEP uptake among MSM after Bayesian spatio‐temporal ecological adjustment

#### 3.2.1 Univariable models

Results estimated by the null model are summarized in Supplements S6–S8. Similar to the null model, we did not find a significant trend in the posterior relative probability of PrEP uptake among MSM over the selected periods (Table 2) for all univariable models. We found that all selected psychosocial and demographical regional determinants of PrEP uptake among MSM, except intention to use PrEP, had a significant impact on PrEP uptake in France. For example, our findings suggested for each 10% increase of sufficient PrEP knowledge among the HIV‐negative MSM population in France, the posterior relative probability of the overall PrEP uptake among MSM increased by 72.1%. In addition, we found that at the regional level, low education is associated with a lower likelihood of PrEP uptake. For each 10% increase in low education among the HIV‐negative MSM population, the regional posterior relative probability of PrEP uptake decreases by 55.1%. For details of each univariable model, see Table 2.

**Table 2:** Models summary of Bayesian spatio‐temporal analysis of PrEP uptake in France, 2016_S1‐2021_S1

		PrEP uptake among MSM in mainland France				
Models	Covariates	Coefficient	95% CrI	Temporal period	95% CrI	DIC
**Spatio‐temporal null model**	Intercept	–0.027	(–0.174;0.119)	–0.001	(–0.021;0.018)	6774.47
**Spatio‐tempotal Univariable models**	PrEP use intention (%)^a^	0.074	(–0.159;0.306)	–0.001	(–0.0021;0.018)	6728.67
	**PrEP sufficient knowledge (%)**	5.429	(5.270;5.589)	0.005	(–0.012;0.023)	5619.95
	**Diagnosed with syphilis, gonorrhoea or chlamydia in the previous 5 years (%)** ^b^	2.592	(2.446;2.737)	0.009	(–0.010;0.037)	6746.71
	**Always CAI with non‐steady male partners (%)**	–14.842	(–15.308;–14.377)	0.014	(–0.007;0.035)	5983.68
	**Reported non‐steady male CAI partners (%)**	–6.626	(–6.851;–6.402)	0.012	(–0.003;0.028)	6258.86
	**Having non‐steady partners with diagnosed HIV (%)**	13.765	(13.319;14.211)	–0.001	(–0.009;0.011)	5655.59
	**Having steady partners living with diagnosed HIV (%)**	5.568	(5.217;5.919)	–0.001	(–0.016;0.014)	6677.06
	**Chemsex** * **in the previous 12 months (%)**	1.851	(1.562;2.141)	–0.001	(–0.018;0.016)	6628.86
	**Chemsex with multiple partners in the previous 12 months (%)**	4.939	(4.591;5.938)	–0.002	(–0.018;0.016)	6360.22
	**Low education (%)** ^c^	–8.009	(–6.278;–7.740)	0.000	(–0.028;0.029)	6088.22
	**Unemployment (%)**	2.423	(2.056;2.789)	0.000	(–0.021;0.021)	6462.55
	**Struggling on current income (%)**	–6.159	(–6.443;–5.876)	0.002	(–0.009;0.014)	7064.20
**Spatio‐temporal multivariable final model**	PrEP use intention (%)^a^	9.146	(–3.842;22.124)	**0.005**	**(0.001;0.09)**	1473.12
	**PrEP sufficient knowledge (%)**	66.529	(55.401;77.647)			
	**Diagnosed with syphilis, gonorrhoea or chlamydia in the previous 5 years (%)** **^b^**	–18.084	(–26.242;–9.933)			
	**Always CAI with non‐steady partners (%)**	–57.739	(–72.640;–42.851)			
	Reported non‐steady male CAI partners (%)	–1.484	(–29.141;26.161)			
	**Having non‐steady partners with diagnosed HIV (%)**	21.278	(6.474;36.070)			
	**Having steady partners living with diagnosed HIV (%)**	–35.983	(–38.147;–33.827)			
	**Chemsex in the previous 12 months (%)**	58.012	(46.791;69.224)			
	Chemsex with multiple partners in the previous 12 months (%)	–34.322	(–77.786;9.106)			
	**Low education (%)** ^c^	99.986	(83.736;116.223)			
	**Unemployment (%)**	–63.350	(–70.920;–55.786)			
	**Struggling on current income (%)**	17.592	(14.586;20.688)			

Abbreviations: CrI, credible interval; DIC, deviance information criterion; ICC, intra‐class correlation.

^a^ Defined as being quite‐ and very‐likely to use PrEP when PrEP is available and affordable.

^b^ Data retrieved from EMIS‐2017 based on non‐French participants.

^c^ Defined as less than 3 years of education beyond 16 years of age.

* Chemsex, use of stimulant drugs (ecstasy/MDMA, cocaine, amphetamine, crystal methamphetamine, mephedrone or ketamine) to make sex more intense or last longer.

#### 3.2.2 Multivariable final model

After model selection, the model with all selected regional determinants (final model) had the smallest DIC, which indicated that the estimated value of the prevalence and posterior relative probability of PrEP uptake among MSM indicated by this model inference was the best match compared to other models. According to this final model, we found a significantly increasing temporal trend of PrEP uptake for the selected period (0.005 [95% CrI 0.001;0.009], Table 2), which indicated that an average 0.5% increase in the posterior relative probability of PrEP uptake was estimated for the periods from 2016_S1 to 2021_S1.

**Figure 3:**
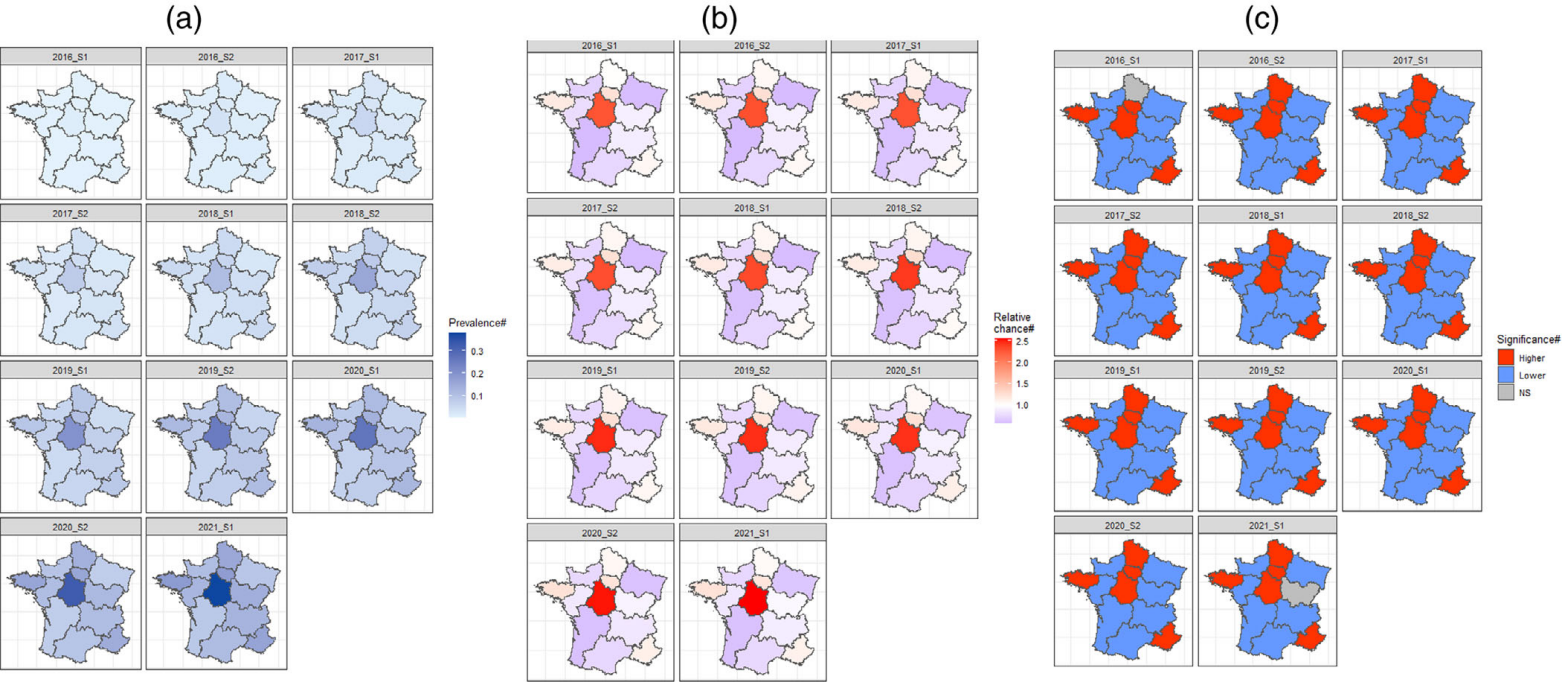
Choropleth map of the (a) estimated posterior prevalence of PrEP uptake among MSM; (b) estimated posterior relative probability of PrEP uptake among MSM; (c) estimated significance of the relative probability of PrEP uptake compared to the overall probability in mainland France, by Bayesian spatio‐temporal ecological modelling (final model) by regions in mainland France, 2016_S1‐2021_S1. Notes: Higher, higher‐than‐average (relative probability >1); Lower, lower‐than‐average (relative probability <1); NS, not significant. # Indicated estimate based on the spatio‐temporal ecological final model. Detailed information can be found in Table S8.

**Figure 4:**
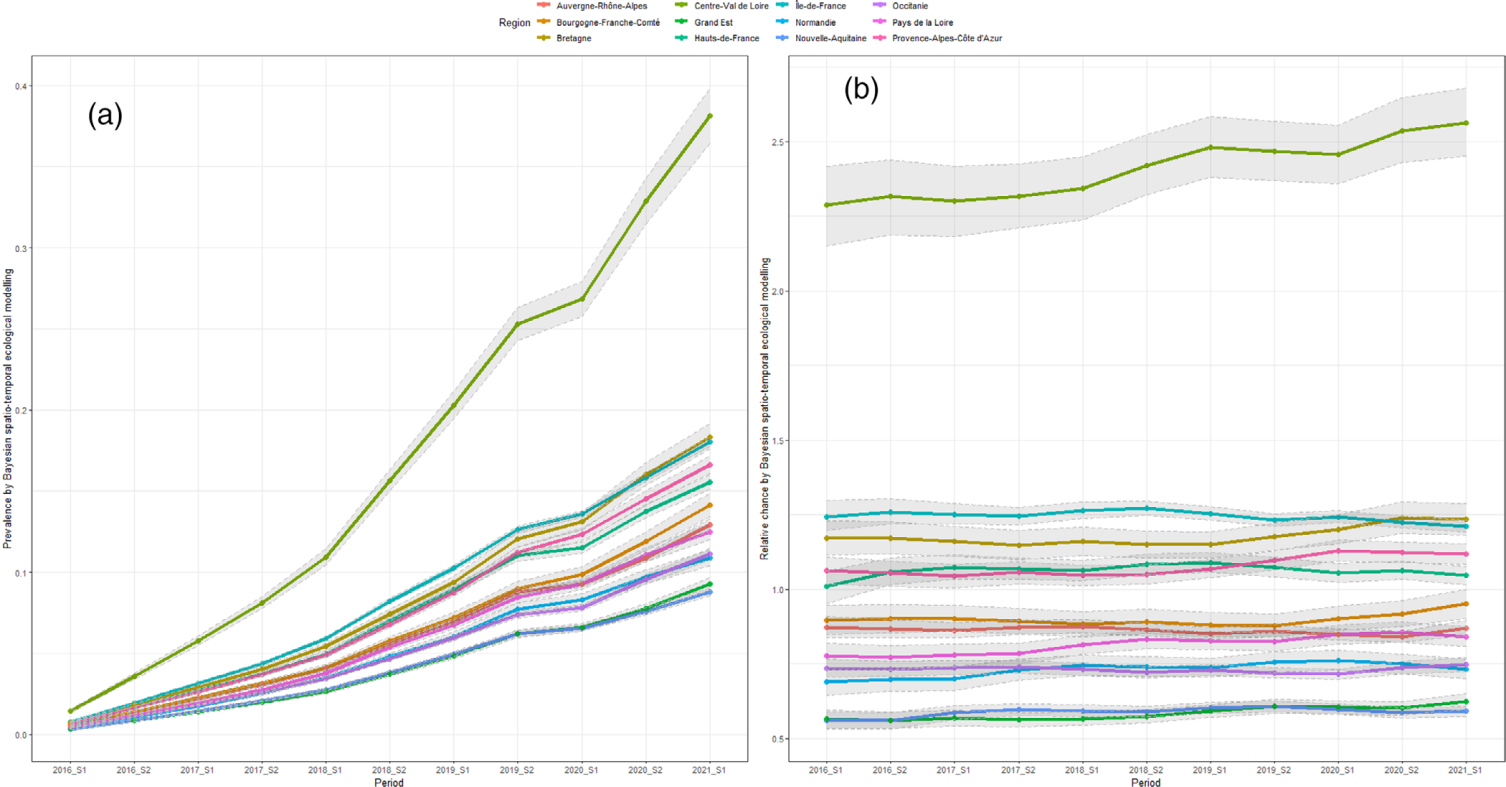
Change of (a) relative probability and (b) prevalence of PrEP uptake among MSM by regions by Bayesian spatio‐temporal ecological final model, mainland France, 2016_S1‐2021_S1.

For the posterior prevalence and relative probability of the overall PrEP uptake, Figure 3 presents the posterior spatial distributions, with information on regions with significant higher‐/lower‐/non‐significant‐posterior relative probability of PrEP uptake (Figure 3c) over the selected periods, and Figure 4 presents a detailed time trend of the (a) posterior relative probability and (b) posterior prevalence of the PrEP uptake among MSM in France. We observed that the overall posterior prevalence has been steadily increasing since the introduction of formal access to PrEP in 2016 and ranged from 8.8% (95% CrI 8.5%;90%) in Nouvelle‐Aquitaine to 38.2% [95% CrI 36.5%;39.9%] in Centre‐Val‐de‐Loire in 2021_S1 (Figure 3a and Supplement S8). On the other hand, the posterior relative probability of PrEP uptake among MSM remained stable compared to the null model's estimation (Supplements S6 and S7). In 2021_S1, compared to the national‐average‐probability of PrEP uptake, the regional posterior relative probability of PrEP uptake ranged from 0.6 [0.57;0.61] in Nouvelle‐Aquitaine to 2.6 [2.46;2.69] in Centre‐Val‐de‐Loire (Figure 3b, c and Supplement S8).

In addition, we observed that both the posterior prevalence and relative probability of the overall PrEP uptake among MSM were much higher in Centre‐Val‐de‐Loire than in other regions (Figures 3 and 4). We also observed a sudden drop for most of the regions in 2020_S1, with the onset of the COVID‐19 pandemic, compared to the previous 2019_S2, and an increase since 2020_S2 based on the final model (Figure 4). However, in terms of the relative probability of PrEP uptake among MSM, most regions across France remained stable since the formal introduction (Figure 4b). More details per region and per period can be found in Table S8. Results and information for the posterior prevalence and relative probability of the new PrEP uptake can be found in Supplements S9–S13.

## 4 DISCUSSION

Using EMIS‐2017 data, national HIV surveillance and national pharmaco‐epidemiology PrEP surveillance data, we applied Bayesian spatial and spatio‐temporal modelling analysis to estimate the regional number of HIV‐negative and PrEP‐eligible MSM, and the posterior prevalence and relative probability of the overall and the new PrEP uptake across France from 2016_S1 to 2021_S1. This, to our knowledge, is the first analysis of its kind. Our analyses revealed substantial regional heterogeneity among HIV‐negative and PrEP‐eligible MSM, as well as a spatio‐temporally heterogeneous distribution of PrEP uptake in France.

### 4.1 Bayesian spatial analysis as a novel method to estimate MSM population size and PrEP eligibility

Robustly estimating the MSM population size is an often unattained prerequisite for public health interventions. Our results provided information on MSM living in France for each region, which delivered insights into HIV surveillance and prevention from a localized perspective for the first time. Our overall estimated HIV‐negative MSM proportion (Table 1) was much lower than the overall proportion of adult MSM estimated by Marcus et al. [13]. Potentially, this is a consequence of the approach using HIV incidence data as a spatial proxy—naturally, MSM with HIV (MSMHIV) are, therefore, excluded in our estimation but still contribute to the sizable MSMHIV population in France overall. Therefore, our results cannot be interpreted as an estimation of the regional MSM population, but should be applied to the HIV‐negative MSM population only.

Contrary to previous studies assuming the national MSM proportion among adult males based on the French national random probability survey of sexual behaviours (CSF) from 2008 [31], our Bayesian approach does not require assumptions on set proportions of MSM among adult males, which allows for more uncertainties during the modelling process. Also, our Bayesian approach smoothened the estimations based on the overall spatial structure [15]. This allowed us to estimate random effects and random noises based on how one region may impact another region under the assumption that residents share more similar characteristics with neighbouring regions than with more distant geographical areas. We thus considered our Bayesian spatial analysis, as a novel method to estimate the HIV‐negative MSM population size, feasible and robust, not only in high‐resource settings like in France, but also in resource‐constrained settings. Our approach can be based on survey‐ and surveillance‐based data of newly diagnosed HIV among MSM, and does not require more complex and potentially more costly general population‐based survey such as CSF [31] to estimate the MSM proportion among adult males. In turn, scarce HIV prevention resources can be targeted more efficiently.

In addition, using EMIS‐2017 data, we further estimated the number of PrEP‐eligible MSM regionally across France. We acknowledge that, compared to the French PrEP guideline [26] and the estimations by Annequin et al. [8], our eligibility criteria also included bacterial STI diagnosis in the preceding 5 years. Therefore, our estimated proportions are higher than the estimations by Annequin et al. (around 35% nationwide in France). However, given the sexual health profiles of men who acquire sexually transmitted infections (STIs) and the established links to HIV [32, 33], the inclusion of STI histories is useful to estimate PrEP eligibility under financially constrained conditions (e.g. when PrEP cannot be given to all those who request it).

### 4.2 Spatio‐temporal distribution of PrEP uptake among MSM in mainland France

Based on our final model, we observed a spatially heterogenous PrEP uptake among MSM across France. In general, regions with bigger urban areas have higher‐than‐average PrEP uptake, such as Île‐de‐France (Paris) and Provence‐Alpes‐Côte d'Azur (Marseille and Nice). It was expected that Île‐de‐France had a consistently higher‐than‐average prevalence and uptake compared to other regions, as Île‐de‐France has the highest MSM population density (Table 1). In turn, this was reflected in the PrEP uptake prevalence and probability. Yet, it was unexpected that Centre‐Val‐de‐Loire, a region which is considered “rural France,” has the highest prevalence and the probability of PrEP uptake as an outlier (Figure 3). One reason may be the much smaller HIV‐negative and eligible MSM populations (Table 1) with relatively more PrEP deliveries [9]. Although our Bayesian spatio‐temporal ecological model has smoothened the estimated values, such a ceiling effect cannot be fully avoided. Also, the close proximity to Île‐de‐France may explain our findings, due to commuter effects. However, our data sources are insufficient to provide evidence for such mobility, but could be alleviated by qualitative evidence. In sum, unravelling these estimates of PrEP uptake differences across France helps to improve current PrEP delivery strategies and implementation programmes. HIV prevention services and public health efforts, including but not limited to PrEP provision and delivery, should be allocated to the less urbanized regions with a lower probability of PrEP uptake. Such structural geographical inequality on an ecological level is one of the key barriers to HIV elimination [34].

When comparing the capacity to benefit from PrEP and the actual uptake (Figures 2b and 3a, b), we found that PrEP delivery in France is appropriately focussed. For example, Hauts‐de‐France has the highest proportion of MSM intending to use PrEP according to EMIS‐2017 data (Figure 2b). Since 2016_S2, this region had a higher‐than‐average probability of PrEP uptake, which reflects successful delivery (Figure 3c). This needs–delivery relationship was also reflected by our univariable model in that a higher regional PrEP uptake is associated with a higher PrEP intention (Table 2). However, we stress that the overall PrEP uptake among eligible MSM remained relatively low (in most regions, the prevalence of the overall PrEP uptake in 2021_S1 was lower than 20%, final model, Supplement S8), with an exception of Centre‐Val‐de‐Loire. Even though our estimates are not devoid of being conservative and potentially underestimated (see Limitations), we concluded that there is a gap in current PrEP uptake, especially when the estimated increasing probability of new PrEP uptake among MSM of 0.8% on average per period (Supplements S9–S13) does not increase substantially. According to previous studies, a PrEP coverage of 30–50% is needed to achieve a 10‐year reduction in HIV incidence of 25% [35, 36].

We found a slow but significant increase in PrEP uptake in France overall, although this temporal trend was significant in both spatio‐temporal null and ecological univariable models. One reason why our models failed to pick up the overall temporal trend may be the sharp drop in PrEP delivery and uptake in 2020_S1 due to COVID‐19 lockdowns, suggesting that some MSM discontinued using PrEP definitively or temporarily [9, 37]. These results reflect the interaction of the HIV epidemic and the COVID‐19 pandemic discussed by a previous study [37], whereby the probability of PrEP uptake decreased due to decreased sexual activities, social distancing or lockdowns.

### 4.3 Strengths and limitations

Our study has two key strengths. First, the analysis was carried out based on national pharmaco‐epidemiology PrEP surveillance data since the beginning of PrEP introduction in France, therefore, our estimations for PrEP uptake among MSM are both comprehensive and not subject to self‐report information biases. Second, the application of the ecological modelling technique together with the Bayesian spatio‐temporal analysis can be considered another major strength in this study. This application allowed us to investigate the variations of PrEP use together with other regional determinants of PrEP uptake, while the space–time components included in the null model could not pick up these additional associations and noise. Consequently, this approach increases certainty when interpreting the results and tailoring interventions for PrEP use among MSM in France. We consider our results as robust and valuable for PrEP‐/HIV‐related policies and prevention strategies to assist in reaching the 2030 elimination goal [1].

To be able to conduct our integration of different data sources, several trade‐offs must be accepted. One major limitation in our MSM population‐size estimation analyses is the retrieved overall MSM population from 2008 [11]. Given an increased social acceptance towards MSM [38], the retrieved MSM population size is probably too conservative. A second challenge is the use of different data sources, surveillance and survey data, which requires that all data used are at the same level (e.g. adjusted vs. unadjusted for undiagnosed HIV cases as discussed and modelled by Marty et al. [12]). This information is not available for all data sources, time points and regions. We prioritized homogeneity over incidentally higher data quality, thus our estimations for HIV‐negative or PrEP‐eligible MSM populations can be seen as underestimates. Third, we applied the EMIS‐2017‐based psychosocial determinants for all periods included, assuming these determinants were stable and comparable between 2016S1 and 2021S1. Given the established increasing trends of some of the included determinants, such as the STIs incidence [39], CAI [40] and chemsex [41], our estimations may be underestimated. Finally, due to a wording issue in the French‐language version (possible confound between STI testing and diagnosis) of EMIS‐2017, we extrapolated the proportion of MSM with an STI diagnosis in the preceding 5‐year from participants in France completing the survey in languages other than French [27]. Consequently, our estimation of MSM with diagnosed bacterial STIs per region may be biased, as there may be a regional difference in STI history between the French immigrant and non‐immigrant based on evidence from EMIS‐2010 [42].

## 5 CONCLUSIONS

In conclusion, our study demonstrates that the population of HIV‐negative MSM in France is diverse, as is the subgroup eligible for formal PrEP access. We describe a temporally increasing and spatially heterogenous distribution of PrEP uptake overall as well as among new users. Regions that would benefit from greater tailoring and delivery efforts are identified. Our findings can aid national and local planning of PrEP services. We, therefore, suggest tailored public health efforts to increase PrEP coverage among the eligible MSM population. Since June 2021, the opportunity to prescribe PrEP has been extended to all prescribing medical doctors in France (including general practitioners) as a measure to improve uptake. We also suggest that PrEP provision and delivery should go beyond the budget‐driven “very‐high‐risk group” approach and develop towards tailored, but comprehensive HIV prevention packages available to all MSM.

## COMPETING INTERESTS

The authors declare no competing interests.

## AUTHORS’ CONTRIBUTIONS

HW, DVDV and KJJ conceptualized this research; HW, JMM, RDS, AJS, FH and KJJ collected the data for this research; HW analysed the data; HW and KJJ drafted the manuscript; all authors critically revised the manuscript for intellectual content; AJS and FH edited the manuscript. All authors read and approved the final version of the manuscript.

## FUNDING

There was no funding source for this study.

## Supporting information


**Table S1** Data sources and functions.
**Figure S2** Mainland France spatial connectivity between regions.
**File S3** Estimation of the size of MSM population that is HIV‐negative / is eligible for PrEP in France.
**File S4** Model assumptions and parameter appointments for the spatio‐temporal analysis of the relative probability of PrEP uptake.
**Table S5** Regional characteristics for determinants of PrEP in Mainland France.
**File S6** Prevalence and relative probability of PrEP uptake among MSM after Bayesian spatio‐temporal adjustment (null model).
**Figure S7** Choropleth map of the a) Estimated prevalence of the overall PrEP uptake among MSM; b) Estimated relative probability of the overall PrEP uptake among MSM; c) Estimated significance of the relative probability of the overall PrEP uptake compared to the overall probability in Mainland France, by Bayesian spatio‐temporal modelling (null model) by regions in Mainland France, 2016_S1‐2021_S1.

## Data Availability

The data that support the findings of this study are available from the corresponding author upon reasonable request.
