# Supplementary material for: Spatio‐temporal changes in pre‐exposure prophylaxis uptake among MSM in mainland France between 2016 and 2021: a Bayesian small area approach with MSM population estimation
Source: J Int AIDS Soc. 2023 May 23;26(5):e26089. doi: 10.1002/jia2.26089 (PMC10206410; doi:10.1002/jia2.26089)
Supplement: Supplementary file 1 — Table S1 Data sources and functions. Figure S2 Mainland France spatial connectivity between regions. File S3 Estimation of the size of MSM population that is HIV‐negative / is eligible for PrEP in France. File S4 Model assumptions and parameter appointments for the spatio‐temporal analysis of the relative probability of PrEP uptake. Table S5 Regional characteristics for determinants of PrEP in Mainland France. File S6 Prevalence and relative probability of PrEP uptake among MSM after Bayesian spatio‐temporal adjustment (null model). Figure S7 Choropleth map of the a) Estimated prevalence of the overall PrEP uptake among MSM; b) Estimated relative probability of the overall PrEP uptake among MSM; c) Estimated significance of the relative probability of the overall PrEP uptake compared to the overall probability in Mainland France, by Bayesian spatio‐temporal modelling (null model) by regions in Mainland France, 2016_S1‐2021_S1. [file JIA2-26-e26089-s001.docx]

**Supplementary Material**

**Spatio-temporal changes in pre-preexposure prophylaxis (PrEP) uptake among MSM in mainland France between 2016 and 2021: A Bayesian small area approach with MSM population estimation**

Haoyi Wang^1,2§^, Jean-Michel Molina^3^, Rosemary Dray-Spira^4^, Axel J. Schmidt^5^, Ford Hickson^5^, David van de Vijver^2^, Kai J. Jonas^1§^

1 Department of Work and Social Psychology, Maastricht University, Maastricht, the Netherlands

2 Viroscience department, Erasmus Medical Centre, Rotterdam, The Netherlands

3 Department of Infectious Diseases, Hôpital Saint-Louis, University of Paris Cité, Paris, France

4 EPI-PHARE, French National Agency for Medicines and Health Products Safety (ANSM) and French National Health Insurance (CNAM), Saint-Denis, France

5 Sigma Research, London School of Hygiene and Tropical Medicine, London, United Kingdom

^§^Correspondence to: Haoyi Wang and Kai J Jonas,

Department of Work and Social Psychology, Maastricht University, Maastricht, 6200ER, the Netherlands

Phone: +31 43 38 84068

Email: haoyi.wang@maastrichtuniversity.nl, kai.jonas@maastrichtuniversity.nl

# Table S1 Data sources and functions

| Data source | Data | Data level | Functions | Reference |
| --- | --- | --- | --- | --- |
| EMIS-2017 French sub-sample | - 2017 newly-diagnosed HIV survey-based self-reported data;  - Self-reported data on psycho-social and behavioural determinants related to PrEP use including: PrEP use intention; PrEP knowledge; being diagnosed with syphilis, gonorrhoea, or chlamydia in the preceding five years; engaging in condomless anal intercourse (CAI) with non-steady male partners; reporting male non-steady CAI partners; chemsex; having steady or non-steady partners with diagnosed HIV  - Self-reported data on demographic determinants including: low educarion; unemployment and struggling on present income | Data was provided at individual level. Data were further aggregated to the French metropolitan regional level based on the provided postal codes. | 1. The 2017 newly-diagnosed HIV survey-based self-reported data was used to estimate the regional risk of the newly-diagnosed HIV in 2017 in mainland France through a Bayesian spatial analysis.  2. The Self-reported data on psycho-social and behavioural determinants related to PrEP use and sociodemographic demterminants were used in the Bayesain spatio-temporal analysis to provide robust estimation on PrEP use in France on the regional level together with revealing co-variation to account for the space-related associations. | [1] |
| Santé Publique France | 2017 newly-diagnosed HIV surveillance unadjusted data | Data was provided at the French metropolitan regional level. | Based on the results from the the regional risk of the newly-diagnosed HIV in 2017 using the self-reported data from EMIS-2017, the surveillance data from Santé Publique France was used to estimate the HIV-negative MSM and PrEP-eligible MSM population. | [2] |
| EPI-PHARE | Pharmaceutical PrEP delivery data for the years 2016–21 | Data was provided at the French metropolitan regional level. | Pharmaceutical PrEP delivery data for the years 2016–21 was used for the spatio-tempotal analysis of the PrEP use from 2016-21 in France on the regional level. | [3] |

# Figure S2 Mainland France spatial connectivity between regions.

**
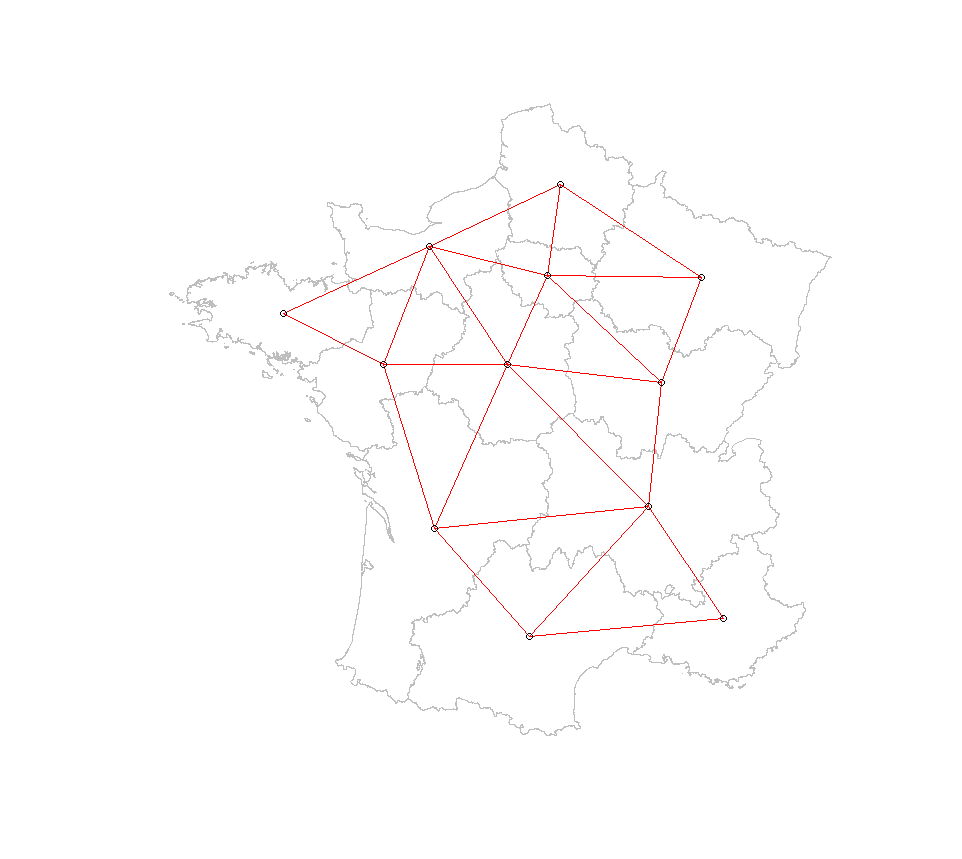
**

^Notes: For names and further information for each region in Mainland France, please see: https://about-france.com/regions.htm^

## File S3 Estimation of the size of MSM population that is HIV-negative / is eligible for PrEP in France

For the estimation of the eligible MSM population for PrEP uptake across France, we first modelled the posterior relative risk (RR) of the newly-diagnosed HIV per region using data from EMIS-2017 using Bayesian spatial analysis, assuming the posterior RRs are comparable to the true RR in reality, which was presented and discussed by Wang et al. [4]. To only include MSM at risk for acquiring HIV in 2017 with the EMIS-2017 data, we first exclude 63 participants who did not answer the question if they had ever been diagnosed with HIV, and 1,168 participants who were diagnosed with HIV more than 12 months ago, leaving n=9,154 in the final sample.

For the counted outcome (newly-diagnosed HIV), we assumed that the observed newly diagnosed HIV cases in EMIS-2017 in each region to follow Poisson distribution with mean $E_{i}\theta_{i}$, where $E_{i}$ is the expected number and $\theta_{i}$ is the RR in each region $i$:

$$Y_{i}\sim Po(E_{i}\theta_{i}),\text{ }i=1,\ldots,N,$$

We thus can define the posterior RR on the logarithmic scale:

$$\log\left( \theta_{i} \right)=\alpha+u_{i}+v_{i}$$

$$u_{i}+v_{i}\boldsymbol{=b}=\frac{1}{\sqrt{\tau_{b}}}(\sqrt{1-\phi}\boldsymbol{v}_{\mathbf{*}}+\sqrt{\phi}\boldsymbol{u}_{\mathbf{*}})$$

Where $\alpha$ represents the overall risk of newly-diagnosed HIV in the France, $\mathcal{ui}$ is a random effect on area $\mathcal{i}$ which is used to model spatial dependence between the RR, and $\mathcal{vi}$ represents other unstructured noise which follow a distribution of $v_{i}\sim N\left( 0,\sigma_{v}^{2} \right)$. [5]. denotes the random effects, $\tau_{b}>0$ is a marginal precision parameter contribution from spatial term $\boldsymbol{u}_{\mathbf{*}}$ and random effect $\boldsymbol{v}_{\mathbf{*}}$, and the fraction of this variance explained by the from spatial term $\boldsymbol{u}_{\mathbf{*}}$ and random effect $\boldsymbol{v}_{\mathbf{*}}$ are the mixing parameter $0\leq\phi\leq1$.

To define the PC prior, we used the probability statement $P((1/\sqrt{\tau_{b}})>U)=\alpha$. Based on the rule of thumb by Simpson et al. [5], we set $U=0.5/0.31$ and $a=0.01$. We then defined the prior for the mixing parameter $\phi$ as $P(\phi<0.5)=2/3$, which assumed that the unstructured random effect accounts for more of the variability than the spatially structured effects [6].

We then estimated the HIV incidence in France in 2017 using the HIV surveillance newly-diagnosed data and the estimated total MSM population in France by Le Vu et al. (2010) [7], which has been adapted by Ndawinz et al. (2015) for the HIV-negative MSM population [8], to estimate a nationwide HIV incidence in 2017. Next, we estimated the HIV-negative MSM population per region ($N_{i}$) across France based on the posterior RR ($\theta_{i}$) estimated from the model, using the overall HIV incidence ($I$) and the HIV surveillance data ($O_{i}$) as the spatial-proxies.

$$N_{i}=\frac{O_{i}}{\theta_{i}I}, i=1,\ldots,n,$$

To account for the eligibility of PrEP prescription, we further estimated the proportion ($P_{i}$) of the eligible population per region using the EMIS-2017 data. In line with the French PrEP guideline [9] and a previous study [10], among HIV-negative MSM, we first included men who ever had sex with other men within the previous12 months as a must-meet criteria. Then, other epidemiological and psycho-social criteria were considered jointly, including ever-diagnosed any bacterial sexually transmitted infections (STIs, additional criteria to the French guideline), having partners living with HIV, ever having condomless anal intercourses (CAIs) with non-steady partners, and ever have chemsex. Given the potential survey effects from EMIS-2017 discussed in an earlier study [11], we controlled the STI variables by language and by data quality, more information can be found in the Limitation section. Lastly, we estimated the size of eligible MSM population per region ($E_{i}$) for the PrEP distribution analyses, assuming the MSM population size is stable over the time.

$$E_{i}= N_{i}\times P_{i}=\frac{O_{i}}{\theta_{i}I}\times P_{i}, i=1,\ldots,n,$$

## File S4 Model assumptions and parameter appointments for the spatio-temporal analysis of the relative probability of PrEP uptake

Similar to the spatial analysis of the relative risk of HIV incidence, we applied the same assumptions for the counted outcome (PrEP uptake), with the same parameters’ appointments to the spatial structure. Additionally, we added a temporal dimension into the model with a spatio-temporal interaction, so the model was formed as:

$$\log(\theta_{i})=\alpha+\boldsymbol{b}+\left( \beta+\delta_{i} \right) t_{j},$$

where $\left( \beta+\delta_{i} \right) t_{j}$ stands for the space-time interaction term.

In in spatio-temporal ecological analysis, we first applied the univariable models which only include one of the selected determinants:

$$\log\left( \theta_{i} \right)=\alpha+\beta_{1}d_{i}+\boldsymbol{b}+\left( \beta+\delta_{i} \right) t_{j}$$

where $d_{i}$ represent the one of the deteminants selected in this study and $\beta_{1}$ is the coefficients for the vector $d_{i}$. In the final model, we included all the significant determinants indicated by the univariate models to evaluate impact on PrEP uptake among MSM in Mainland France:

$$\log\left( \theta_{i} \right)=\boldsymbol{d}_{i}\boldsymbol{\beta}+\boldsymbol{b}+\left( \beta+\delta_{i} \right) t_{j}$$

$$\boldsymbol{d}_{i}=(1,d_{i1},\ldots,d_{ip})$$

$$\boldsymbol{\beta}=(\beta_{0},\beta_{1},\ldots,\beta_{p})^{'}$$

## Table S5. Regional characteristics for determinants of PrEP in Mainland France

| Determinants | **Regions in France Mainland** | | | | | | | | | | | |
| --- | --- | --- | --- | --- | --- | --- | --- | --- | --- | --- | --- | --- |
|  | Auvergne-Rhône-Alpes | Bourgogne-Franche-Comté | Bretagne | Centre-Val de Loire | Grand Est | Hauts-de-France | Île-de-France | Normandie | Nouvelle-Aquitaine | Occitanie | Pays de la Loire | Provence-Alpes-Côte d'Azur |
| Sufficient PrEP knowledge | 0,28 | 0,20 | 0,24 | 0,28 | 0,25 | 0,30 | 0,36 | 0,24 | 0,27 | 0,29 | 0,28 | 0,30 |
| Diagnosed with syphilis, gonorrhoea, or chlamydia in the previous 5 years^*^ | 0,14 | 0,00 | 0,13 | 0,00 | 0,10 | 0,15 | 0,13 | 0,08 | 0,05 | 0,115 | 0,00 | 0,14 |
| Always CAI with non-steady partners | 0,05 | 0,06 | 0,02 | 0,08 | 0,08 | 0,06 | 0,04 | 0,07 | 0,07 | 0,05 | 0,08 | 0,06 |
| Reported non-steady male CAI partners (%) | 0,55 | 0,55 | 0,52 | 0,60 | 0,57 | 0,58 | 0,51 | 0,57 | 0,60 | 0,54 | 0,55 | 0,55 |
| Having non-steady partners with diagnosed HIV (%) | 0,11 | 0,08 | NA | 0,09 | 0,09 | 0,11 | 0,13 | 0,08 | 0,13 | 0,11 | 0,09 | 0,12 |
| Having steady partners living with diagnosed HIV (%) | 0,07 | NA | NA | 0,08 | 0,06 | 0,07 | 0,09 | 0,04 | 0,08 | 0,06 | 0,04 | 0,08 |
| Chemsex* in the previous 12 months (%) | 0,09 | 0,05 | 0,07 | 0,11 | 0,06 | 0,07 | 0,10 | 0,08 | 0,09 | 0,08 | 0,09 | 0,10 |
| Chemsex with multiple partners in the previous 12 months (%) | 0,02 | NA | NA | NA | NA | NA | 0,04 | NA | 0,04 | NA | NA | 0,04 |
| Low education (%) | 0,10 | 0,15 | 0,15 | 0,12 | 0,14 | 0,09 | 0,06 | 0,11 | 0,11 | 0,12 | 0,11 | 0,14 |
| Unemployment (%) | 0,09 | 0,11 | 0,08 | 0,11 | 0,09 | 0,10 | 0,10 | 0,07 | 0,11 | 0,13 | 0,11 | 0,14 |
| Struggling on current income (%) | 0,15 | 0,15 | 0,17 | 0,17 | 0,16 | 0,20 | 0,12 | 0,18 | 0,15 | 0,16 | 0,15 | 0,15 |

^Notes: Data retrieved from EMIS-2017.^ ^* Data retrieved from non-French language subsample from EMIS-2017. Data are presented in proportion format. NA = not available for that region.^

## File S6 Prevalence and relative probability of PrEP uptake among MSM after Bayesian spatio-temporal adjustment (null model)

After accounting for the spatio-temporal effects based on the spatial structure of France presented in Figure S5 without other regional determinants of PrEP uptake (null model), we found an decreasing but non-significant temporal trend of PrEP uptake among MSM in France for the selected periods, with a coefficient of -0,001(95%CrI -0,021;0,018), which indicated for each later period after 2016S1, the relative probability of PrEP uptake decreased by 0.01%=1-exp(-0.001), details see Table 2. The estimated ICC of the spatial structure was estimated at 0.30, which indicates that around 30% of the observed variance of PrEP uptake among MSM in France can be explained by the unobserved information on the spatial structure.

The posterior prevalence and the posterior relative probability of overall PrEP uptake were heterogenous in France from the selected periods. Figure S7 shows a dynamic overview of the S7-a) prevalence and S7-b) relative probability of PrEP uptake, with information on regions with significant higher-/lower-/non-significant-relative probability of the overall PrEP uptake (Figure 3-c). More details of the overall PrEP uptake for each region each time period can be found in the Supplementary S8 table.


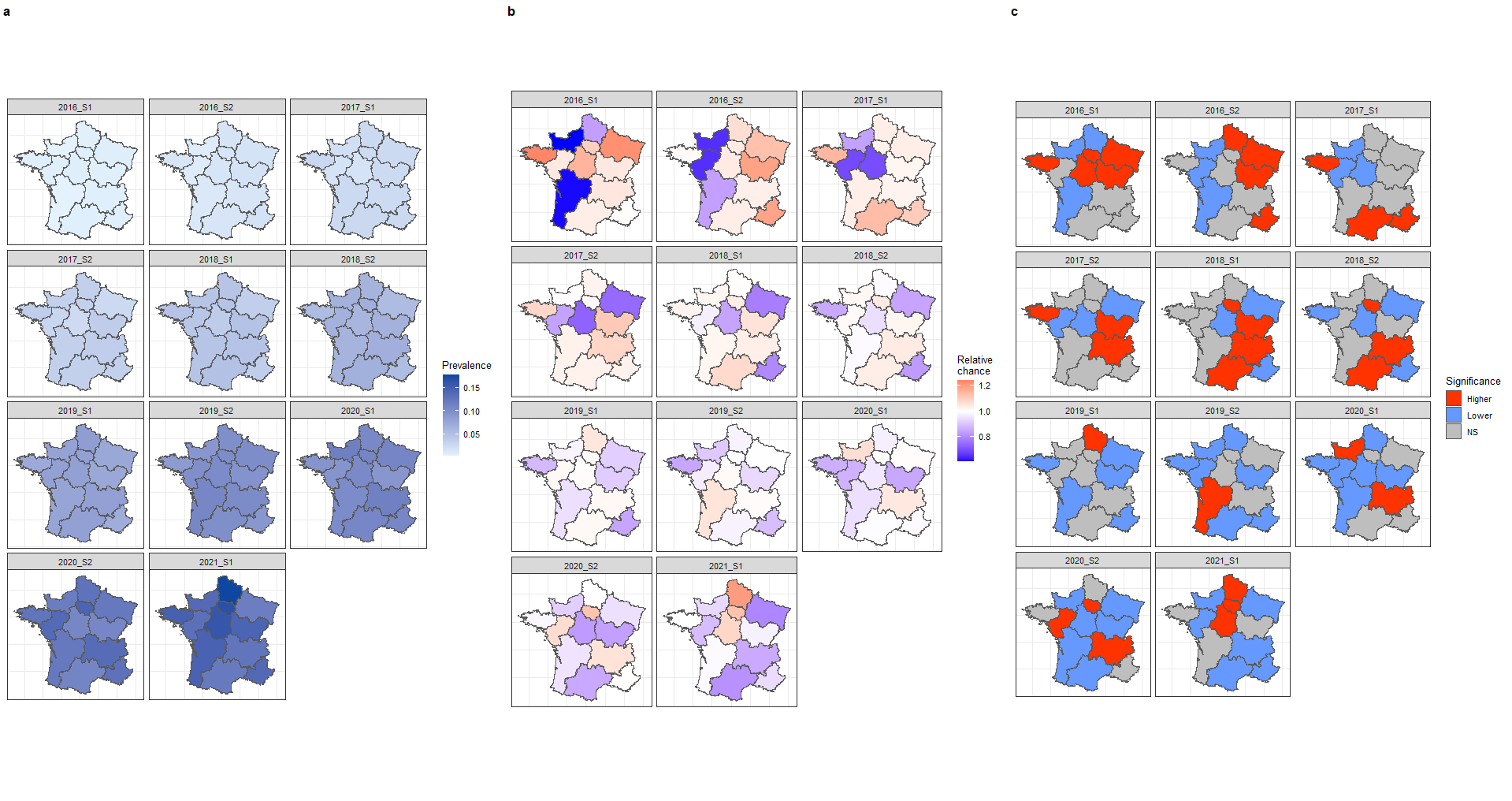
**Figure S7 Choropleth map of the a) Estimated prevalence of the overall PrEP uptake among MSM; b) Estimated relative probability of the overall PrEP uptake among MSM; c) Estimated significance of the relative probability of the overall PrEP uptake compared to the overall probability in Mainland France, by Bayesian spatio-temporal modelling (null model) by regions in Mainland France, 2016_S1-2021_S1.**

^Notes: Higher=higher-than-average (relative probability >1); Lower=lower-than-average (relative probability <1); NS=not significant. Detailed information can be found in Supplementary Table S4.^

## Table S8 Prevalence and relative probability of PrEP uptake: model comparison

| **Period** | **Region** | **Bayesian spatio-temporal null model** | | **Bayesian spatio-temporal final model** | |
| --- | --- | --- | --- | --- | --- |
|  |  | **Relative probability (95%CrI)** | **Prevalence (95%CrI)** | **Relative probability (95%CrI)** | **Prevalence (95%CrI)** |
| 2016  S1 | Auvergne-Rhône-Alpes | 1,049 (0,997;1,103) | 0,007 (0,006;0,007) | 0,871 (0,837;0,909) | 0,006 (0,005;0,006) |
|  | Bourgogne-Franche-Comté | 1,047 (1,001;1,095) | 0,007 (0,006;0,007) | 0,899 (0,852;0,949) | 0,006 (0,005;0,006) |
|  | Bretagne | 1,24 (1,179;1,303) | 0,008 (0,007;0,008) | 1,171 (1,116;1,23) | 0,007 (0,007;0,008) |
|  | Centre-Val de Loire | 1,151 (1,077;1,23) | 0,007 (0,007;0,008) | 2,29 (2,153;2,42) | 0,014 (0,014;0,015) |
|  | Grand Est | 1,221 (1,149;1,297) | 0,008 (0,007;0,008) | 0,566 (0,536;0,594) | 0,004 (0,003;0,004) |
|  | Hauts-de-France | 0,838 (0,777;0,902) | 0,005 (0,005;0,006) | 1,009 (0,952;1,061) | 0,006 (0,006;0,007) |
|  | Île-de-France | 1,098 (1,037;1,16) | 0,007 (0,007;0,007) | 1,244 (1,198;1,299) | 0,008 (0,008;0,008) |
|  | Normandie | 0,609 (0,56;0,66) | 0,004 (0,004;0,004) | 0,692 (0,646;0,735) | 0,004 (0,004;0,005) |
|  | Nouvelle-Aquitaine | 0,614 (0,568;0,662) | 0,004 (0,004;0,004) | 0,561 (0,531;0,589) | 0,004 (0,003;0,004) |
|  | Occitanie | 1,036 (0,989;1,084) | 0,007 (0,006;0,007) | 0,735 (0,707;0,767) | 0,005 (0,004;0,005) |
|  | Pays de la Loire | 1,045 (0,976;1,118) | 0,007 (0,006;0,007) | 0,774 (0,728;0,817) | 0,005 (0,005;0,005) |
|  | Provence-Alpes-Côte d'Azur | 1,005 (0,946;1,066) | 0,006 (0,006;0,007) | 1,064 (1,019;1,108) | 0,007 (0,006;0,007) |
| 2016  S2 | Auvergne-Rhône-Alpes | 1,032 (0,991;1,074) | 0,016 (0,015;0,017) | 0,866 (0,837;0,898) | 0,013 (0,013;0,014) |
|  | Bourgogne-Franche-Comté | 1,186 (1,136;1,237) | 0,018 (0,018;0,019) | 0,903 (0,858;0,951) | 0,014 (0,013;0,015) |
|  | Bretagne | 1,012 (0,962;1,064) | 0,016 (0,015;0,017) | 1,172 (1,119;1,226) | 0,018 (0,017;0,019) |
|  | Centre-Val de Loire | 1,04 (0,979;1,103) | 0,016 (0,015;0,017) | 2,319 (2,19;2,443) | 0,036 (0,034;0,038) |
|  | Grand Est | 1,127 (1,064;1,193) | 0,017 (0,017;0,019) | 0,562 (0,534;0,589) | 0,009 (0,008;0,009) |
|  | Hauts-de-France | 1,065 (1,013;1,119) | 0,017 (0,016;0,017) | 1,059 (1,016;1,107) | 0,016 (0,016;0,017) |
|  | Île-de-France | 1,042 (0,997;1,088) | 0,016 (0,015;0,017) | 1,259 (1,219;1,304) | 0,02 (0,019;0,02) |
|  | Normandie | 0,652 (0,608;0,698) | 0,01 (0,009;0,011) | 0,701 (0,66;0,74) | 0,011 (0,01;0,011) |
|  | Nouvelle-Aquitaine | 0,84 (0,785;0,897) | 0,013 (0,012;0,014) | 0,56 (0,531;0,587) | 0,009 (0,008;0,009) |
|  | Occitanie | 1,036 (0,996;1,076) | 0,016 (0,015;0,017) | 0,732 (0,707;0,759) | 0,011 (0,011;0,012) |
|  | Pays de la Loire | 0,652 (0,605;0,7) | 0,01 (0,009;0,011) | 0,771 (0,727;0,811) | 0,012 (0,011;0,013) |
|  | Provence-Alpes-Côte d'Azur | 1,183 (1,123;1,245) | 0,018 (0,017;0,019) | 1,054 (1,01;1,096) | 0,016 (0,016;0,017) |
| 2017  S1 | Auvergne-Rhône-Alpes | 1,024 (0,99;1,059) | 0,026 (0,025;0,027) | 0,862 (0,836;0,889) | 0,022 (0,021;0,022) |
|  | Bourgogne-Franche-Comté | 1,019 (0,977;1,062) | 0,026 (0,025;0,027) | 0,905 (0,861;0,95) | 0,023 (0,022;0,024) |
|  | Bretagne | 1,146 (1,097;1,196) | 0,029 (0,028;0,03) | 1,161 (1,109;1,212) | 0,029 (0,028;0,03) |
|  | Centre-Val de Loire | 0,697 (0,654;0,742) | 0,017 (0,016;0,019) | 2,305 (2,184;2,421) | 0,058 (0,055;0,061) |
|  | Grand Est | 1,034 (0,982;1,088) | 0,026 (0,025;0,027) | 0,569 (0,542;0,594) | 0,014 (0,014;0,015) |
|  | Hauts-de-France | 1,034 (0,995;1,073) | 0,026 (0,025;0,027) | 1,074 (1,035;1,116) | 0,027 (0,026;0,028) |
|  | Île-de-France | 1,029 (0,994;1,064) | 0,026 (0,025;0,027) | 1,252 (1,219;1,288) | 0,031 (0,031;0,032) |
|  | Normandie | 0,842 (0,793;0,891) | 0,021 (0,02;0,022) | 0,703 (0,663;0,741) | 0,018 (0,017;0,019) |
|  | Nouvelle-Aquitaine | 1,034 (0,989;1,079) | 0,026 (0,025;0,027) | 0,586 (0,564;0,609) | 0,015 (0,014;0,015) |
|  | Occitanie | 1,134 (1,094;1,175) | 0,028 (0,027;0,029) | 0,738 (0,715;0,762) | 0,019 (0,018;0,019) |
|  | Pays de la Loire | 0,693 (0,652;0,735) | 0,017 (0,016;0,018) | 0,778 (0,741;0,814) | 0,02 (0,019;0,02) |
|  | Provence-Alpes-Côte d'Azur | 1,104 (1,051;1,158) | 0,028 (0,026;0,029) | 1,045 (1,004;1,084) | 0,026 (0,025;0,027) |
| 2017  S2 | Auvergne-Rhône-Alpes | 1,085 (1,053;1,117) | 0,038 (0,037;0,039) | 0,872 (0,848;0,897) | 0,031 (0,03;0,031) |
|  | Bourgogne-Franche-Comté | 1,11 (1,072;1,149) | 0,039 (0,038;0,04) | 0,896 (0,853;0,939) | 0,031 (0,03;0,033) |
|  | Bretagne | 1,081 (1,039;1,125) | 0,038 (0,036;0,039) | 1,149 (1,1;1,198) | 0,04 (0,039;0,042) |
|  | Centre-Val de Loire | 0,736 (0,698;0,775) | 0,026 (0,025;0,027) | 2,321 (2,214;2,429) | 0,081 (0,078;0,085) |
|  | Grand Est | 0,746 (0,707;0,787) | 0,026 (0,025;0,028) | 0,565 (0,54;0,588) | 0,02 (0,019;0,021) |
|  | Hauts-de-France | 1,025 (0,995;1,055) | 0,036 (0,035;0,037) | 1,069 (1,033;1,106) | 0,038 (0,036;0,039) |
|  | Île-de-France | 1,014 (0,985;1,043) | 0,036 (0,035;0,037) | 1,246 (1,218;1,276) | 0,044 (0,043;0,045) |
|  | Normandie | 1,004 (0,966;1,041) | 0,035 (0,034;0,037) | 0,731 (0,697;0,767) | 0,026 (0,024;0,027) |
|  | Nouvelle-Aquitaine | 1,026 (0,994;1,06) | 0,036 (0,035;0,037) | 0,596 (0,576;0,617) | 0,021 (0,02;0,022) |
|  | Occitanie | 1,026 (0,993;1,061) | 0,036 (0,035;0,037) | 0,74 (0,719;0,762) | 0,026 (0,025;0,027) |
|  | Pays de la Loire | 0,843 (0,802;0,886) | 0,03 (0,028;0,031) | 0,783 (0,746;0,819) | 0,027 (0,026;0,029) |
|  | Provence-Alpes-Côte d'Azur | 1,029 (0,985;1,074) | 0,036 (0,035;0,038) | 1,057 (1,02;1,094) | 0,037 (0,036;0,038) |
| 2018  S1 | Auvergne-Rhône-Alpes | 1,034 (1,008;1,06) | 0,048 (0,047;0,05) | 0,876 (0,853;0,898) | 0,041 (0,04;0,042) |
|  | Bourgogne-Franche-Comté | 1,059 (1,026;1,092) | 0,05 (0,048;0,051) | 0,885 (0,843;0,927) | 0,041 (0,039;0,043) |
|  | Bretagne | 1,024 (0,988;1,06) | 0,048 (0,046;0,05) | 1,162 (1,114;1,209) | 0,054 (0,052;0,057) |
|  | Centre-Val de Loire | 0,845 (0,81;0,881) | 0,04 (0,038;0,041) | 2,347 (2,24;2,453) | 0,11 (0,105;0,115) |
|  | Grand Est | 0,782 (0,747;0,817) | 0,037 (0,035;0,038) | 0,567 (0,545;0,589) | 0,027 (0,025;0,028) |
|  | Hauts-de-France | 1,002 (0,978;1,027) | 0,047 (0,046;0,048) | 1,064 (1,03;1,098) | 0,05 (0,048;0,051) |
|  | Île-de-France | 1,039 (1,014;1,064) | 0,049 (0,047;0,05) | 1,266 (1,239;1,293) | 0,059 (0,058;0,06) |
|  | Normandie | 1,019 (0,991;1,048) | 0,048 (0,046;0,049) | 0,746 (0,712;0,78) | 0,035 (0,033;0,037) |
|  | Nouvelle-Aquitaine | 1,02 (0,995;1,046) | 0,048 (0,047;0,049) | 0,593 (0,574;0,612) | 0,028 (0,027;0,029) |
|  | Occitanie | 1,075 (1,046;1,105) | 0,05 (0,049;0,052) | 0,732 (0,711;0,752) | 0,034 (0,033;0,035) |
|  | Pays de la Loire | 0,974 (0,943;1,006) | 0,046 (0,044;0,047) | 0,812 (0,781;0,845) | 0,038 (0,037;0,04) |
|  | Provence-Alpes-Côte d'Azur | 0,799 (0,764;0,835) | 0,037 (0,036;0,039) | 1,048 (1,014;1,081) | 0,049 (0,047;0,051) |
| 2018  S2 | Auvergne-Rhône-Alpes | 1,042 (1,02;1,064) | 0,067 (0,066;0,069) | 0,865 (0,844;0,886) | 0,056 (0,055;0,057) |
|  | Bourgogne-Franche-Comté | 1,019 (0,991;1,047) | 0,066 (0,064;0,068) | 0,894 (0,853;0,936) | 0,058 (0,055;0,061) |
|  | Bretagne | 0,855 (0,825;0,886) | 0,055 (0,053;0,057) | 1,15 (1,105;1,195) | 0,074 (0,072;0,077) |
|  | Centre-Val de Loire | 0,946 (0,92;0,972) | 0,061 (0,06;0,063) | 2,424 (2,326;2,526) | 0,157 (0,151;0,163) |
|  | Grand Est | 0,847 (0,818;0,876) | 0,055 (0,053;0,057) | 0,576 (0,553;0,598) | 0,037 (0,036;0,039) |
|  | Hauts-de-France | 0,993 (0,972;1,014) | 0,064 (0,063;0,066) | 1,084 (1,05;1,118) | 0,07 (0,068;0,072) |
|  | Île-de-France | 1,043 (1,022;1,064) | 0,067 (0,066;0,069) | 1,272 (1,248;1,296) | 0,082 (0,081;0,084) |
|  | Normandie | 1,017 (0,994;1,04) | 0,066 (0,064;0,067) | 0,742 (0,709;0,775) | 0,048 (0,046;0,05) |
|  | Nouvelle-Aquitaine | 0,991 (0,97;1,012) | 0,064 (0,063;0,066) | 0,59 (0,572;0,608) | 0,038 (0,037;0,039) |
|  | Occitanie | 1,036 (1,012;1,061) | 0,067 (0,065;0,069) | 0,721 (0,702;0,741) | 0,047 (0,045;0,048) |
|  | Pays de la Loire | 1,012 (0,987;1,037) | 0,066 (0,064;0,067) | 0,83 (0,799;0,862) | 0,054 (0,052;0,056) |
|  | Provence-Alpes-Côte d'Azur | 0,831 (0,801;0,861) | 0,054 (0,052;0,056) | 1,05 (1,019;1,081) | 0,068 (0,066;0,07) |
| 2019  S1 | Auvergne-Rhône-Alpes | 1,015 (0,996;1,034) | 0,083 (0,082;0,085) | 0,851 (0,831;0,871) | 0,07 (0,068;0,071) |
|  | Bourgogne-Franche-Comté | 0,915 (0,89;0,94) | 0,075 (0,073;0,077) | 0,884 (0,844;0,925) | 0,072 (0,069;0,076) |
|  | Bretagne | 0,883 (0,857;0,908) | 0,072 (0,07;0,074) | 1,149 (1,106;1,194) | 0,094 (0,09;0,098) |
|  | Centre-Val de Loire | 1,005 (0,981;1,03) | 0,082 (0,08;0,084) | 2,484 (2,383;2,587) | 0,203 (0,195;0,212) |
|  | Grand Est | 0,918 (0,895;0,941) | 0,075 (0,073;0,077) | 0,592 (0,571;0,614) | 0,048 (0,047;0,05) |
|  | Hauts-de-France | 1,049 (1,029;1,07) | 0,086 (0,084;0,088) | 1,091 (1,058;1,124) | 0,089 (0,087;0,092) |
|  | Île-de-France | 1,011 (0,993;1,029) | 0,083 (0,081;0,084) | 1,255 (1,232;1,278) | 0,103 (0,101;0,105) |
|  | Normandie | 0,981 (0,961;1,001) | 0,08 (0,079;0,082) | 0,738 (0,706;0,771) | 0,06 (0,058;0,063) |
|  | Nouvelle-Aquitaine | 0,949 (0,929;0,969) | 0,078 (0,076;0,079) | 0,603 (0,585;0,622) | 0,049 (0,048;0,051) |
|  | Occitanie | 1,013 (0,992;1,035) | 0,083 (0,081;0,085) | 0,729 (0,71;0,748) | 0,06 (0,058;0,061) |
|  | Pays de la Loire | 1,013 (0,991;1,036) | 0,083 (0,081;0,085) | 0,826 (0,796;0,857) | 0,068 (0,065;0,07) |
|  | Provence-Alpes-Côte d'Azur | 0,849 (0,825;0,873) | 0,069 (0,067;0,071) | 1,07 (1,039;1,101) | 0,088 (0,085;0,09) |
| 2019  S2 | Auvergne-Rhône-Alpes | 1,008 (0,991;1,025) | 0,103 (0,102;0,105) | 0,86 (0,84;0,879) | 0,088 (0,086;0,09) |
|  | Bourgogne-Franche-Comté | 0,937 (0,917;0,958) | 0,096 (0,094;0,098) | 0,881 (0,842;0,921) | 0,09 (0,086;0,094) |
|  | Bretagne | 0,851 (0,83;0,871) | 0,087 (0,085;0,089) | 1,176 (1,131;1,222) | 0,12 (0,116;0,125) |
|  | Centre-Val de Loire | 1,01 (0,986;1,034) | 0,103 (0,101;0,106) | 2,472 (2,374;2,572) | 0,253 (0,243;0,264) |
|  | Grand Est | 0,998 (0,971;1,024) | 0,102 (0,1;0,105) | 0,608 (0,586;0,631) | 0,062 (0,06;0,065) |
|  | Hauts-de-France | 0,978 (0,96;0,996) | 0,1 (0,098;0,102) | 1,075 (1,043;1,107) | 0,11 (0,107;0,113) |
|  | Île-de-France | 0,996 (0,98;1,012) | 0,102 (0,1;0,104) | 1,233 (1,212;1,254) | 0,126 (0,124;0,128) |
|  | Normandie | 0,908 (0,886;0,931) | 0,093 (0,091;0,095) | 0,757 (0,724;0,791) | 0,078 (0,074;0,081) |
|  | Nouvelle-Aquitaine | 1,056 (1,032;1,08) | 0,108 (0,106;0,111) | 0,607 (0,589;0,626) | 0,062 (0,06;0,064) |
|  | Occitanie | 0,979 (0,959;0,998) | 0,1 (0,098;0,102) | 0,72 (0,702;0,738) | 0,074 (0,072;0,076) |
|  | Pays de la Loire | 0,97 (0,949;0,991) | 0,099 (0,097;0,102) | 0,822 (0,792;0,852) | 0,084 (0,081;0,087) |
|  | Provence-Alpes-Côte d'Azur | 0,891 (0,87;0,913) | 0,091 (0,089;0,094) | 1,097 (1,065;1,129) | 0,112 (0,109;0,116) |
| 2020  S1 | Auvergne-Rhône-Alpes | 1,048 (1,032;1,064) | 0,115 (0,113;0,116) | 0,848 (0,829;0,867) | 0,093 (0,091;0,095) |
|  | Bourgogne-Franche-Comté | 0,852 (0,833;0,872) | 0,093 (0,091;0,095) | 0,905 (0,864;0,948) | 0,099 (0,094;0,104) |
|  | Bretagne | 0,865 (0,843;0,887) | 0,095 (0,092;0,097) | 1,201 (1,153;1,249) | 0,131 (0,126;0,137) |
|  | Centre-Val de Loire | 0,96 (0,937;0,984) | 0,105 (0,102;0,108) | 2,459 (2,361;2,559) | 0,269 (0,258;0,28) |
|  | Grand Est | 1,006 (0,979;1,033) | 0,11 (0,107;0,113) | 0,605 (0,583;0,628) | 0,066 (0,064;0,069) |
|  | Hauts-de-France | 0,974 (0,955;0,993) | 0,106 (0,104;0,109) | 1,055 (1,024;1,086) | 0,115 (0,112;0,119) |
|  | Île-de-France | 1,005 (0,989;1,021) | 0,11 (0,108;0,112) | 1,244 (1,224;1,265) | 0,136 (0,134;0,138) |
|  | Normandie | 1,065 (1,033;1,096) | 0,116 (0,113;0,12) | 0,764 (0,73;0,798) | 0,083 (0,08;0,087) |
|  | Nouvelle-Aquitaine | 0,947 (0,924;0,969) | 0,104 (0,101;0,106) | 0,598 (0,581;0,616) | 0,065 (0,063;0,067) |
|  | Occitanie | 0,995 (0,979;1,012) | 0,109 (0,107;0,111) | 0,716 (0,698;0,733) | 0,078 (0,076;0,08) |
|  | Pays de la Loire | 0,869 (0,843;0,895) | 0,095 (0,092;0,098) | 0,846 (0,814;0,879) | 0,093 (0,089;0,096) |
|  | Provence-Alpes-Côte d'Azur | 0,991 (0,961;1,022) | 0,108 (0,105;0,112) | 1,129 (1,094;1,165) | 0,123 (0,12;0,127) |
| 2020  S2 | Auvergne-Rhône-Alpes | 1,058 (1,042;1,073) | 0,137 (0,135;0,139) | 0,841 (0,822;0,859) | 0,109 (0,107;0,111) |
|  | Bourgogne-Franche-Comté | 0,84 (0,816;0,864) | 0,109 (0,106;0,112) | 0,921 (0,877;0,966) | 0,119 (0,114;0,125) |
|  | Bretagne | 0,984 (0,949;1,02) | 0,127 (0,123;0,132) | 1,239 (1,187;1,293) | 0,16 (0,154;0,167) |
|  | Centre-Val de Loire | 0,832 (0,802;0,862) | 0,108 (0,104;0,112) | 2,541 (2,433;2,652) | 0,329 (0,315;0,343) |
|  | Grand Est | 0,949 (0,922;0,976) | 0,123 (0,119;0,126) | 0,602 (0,58;0,625) | 0,078 (0,075;0,081) |
|  | Hauts-de-France | 0,998 (0,979;1,018) | 0,129 (0,127;0,132) | 1,064 (1,033;1,095) | 0,138 (0,134;0,142) |
|  | Île-de-France | 1,123 (1,104;1,141) | 0,145 (0,143;0,148) | 1,225 (1,205;1,245) | 0,159 (0,156;0,161) |
|  | Normandie | 0,918 (0,89;0,946) | 0,119 (0,115;0,123) | 0,752 (0,718;0,786) | 0,097 (0,093;0,102) |
|  | Nouvelle-Aquitaine | 0,953 (0,929;0,978) | 0,123 (0,12;0,127) | 0,586 (0,568;0,604) | 0,076 (0,074;0,078) |
|  | Occitanie | 0,854 (0,833;0,875) | 0,111 (0,108;0,113) | 0,738 (0,717;0,76) | 0,096 (0,093;0,098) |
|  | Pays de la Loire | 1,073 (1,033;1,113) | 0,139 (0,134;0,144) | 0,854 (0,821;0,888) | 0,111 (0,106;0,115) |
|  | Provence-Alpes-Côte d'Azur | 1,003 (0,972;1,035) | 0,13 (0,126;0,134) | 1,124 (1,09;1,159) | 0,146 (0,141;0,15) |
| 2021  S1 | Auvergne-Rhône-Alpes | 0,856 (0,831;0,882) | 0,127 (0,124;0,131) | 0,871 (0,846;0,896) | 0,129 (0,126;0,133) |
|  | Bourgogne-Franche-Comté | 0,977 (0,937;1,019) | 0,145 (0,139;0,152) | 0,953 (0,904;1,003) | 0,142 (0,134;0,149) |
|  | Bretagne | 0,999 (0,963;1,036) | 0,149 (0,143;0,154) | 1,234 (1,182;1,287) | 0,183 (0,176;0,191) |
|  | Centre-Val de Loire | 1,081 (1,032;1,132) | 0,161 (0,153;0,168) | 2,567 (2,455;2,685) | 0,382 (0,365;0,399) |
|  | Grand Est | 0,795 (0,761;0,83) | 0,118 (0,113;0,123) | 0,624 (0,599;0,651) | 0,093 (0,089;0,097) |
|  | Hauts-de-France | 1,201 (1,174;1,228) | 0,179 (0,175;0,183) | 1,046 (1,016;1,077) | 0,156 (0,151;0,16) |
|  | Île-de-France | 1,125 (1,107;1,144) | 0,167 (0,165;0,17) | 1,212 (1,193;1,232) | 0,18 (0,177;0,183) |
|  | Normandie | 0,934 (0,903;0,966) | 0,139 (0,134;0,144) | 0,735 (0,702;0,768) | 0,109 (0,104;0,114) |
|  | Nouvelle-Aquitaine | 0,992 (0,967;1,018) | 0,148 (0,144;0,151) | 0,591 (0,574;0,608) | 0,088 (0,085;0,09) |
|  | Occitanie | 0,815 (0,788;0,842) | 0,121 (0,117;0,125) | 0,748 (0,723;0,772) | 0,111 (0,108;0,115) |
|  | Pays de la Loire | 0,889 (0,855;0,924) | 0,132 (0,127;0,137) | 0,84 (0,807;0,874) | 0,125 (0,12;0,13) |
|  | Provence-Alpes-Côte d'Azur | 0,939 (0,908;0,971) | 0,14 (0,135;0,144) | 1,118 (1,083;1,153) | 0,166 (0,161;0,171) |

^Note: CrI = credible interval^

## File S9 Bayesian spatio-temporal ecological analysis for new PrEP uptake among MSM in France, 2016S1-2021S1

After modelling the spatio-temporal distribution of the prevalence and relative probability of the overall PrEP uptake, we additionally applied the final model, jointly with all selected psycho-social determinants, to model the spatio-temporal distribution of the prevalence and probability of the new PrEP uptake among MSM (the first-time users).

In this analysis, we found a significant increasing overall trend of the new PrEP uptake for the selected period (0.008 [95%CrI 0.002;0.014]), which indicated that an average 0.8% increase of the relative probability of the new PrEP uptake among MSM in France was estimated from 2016S1 to 2021S1, see Table S10 for detailed information of the model.

**Table S10 Spatio-temporal model for the new PrEP uptake among MSM, 2016S1-2021S1**

| **Model** | **Covariates** | **Coefficient** | **95%CrI** | **Temporal period**  **(95%CrI)** | **DIC** | **ICC** |
| --- | --- | --- | --- | --- | --- | --- |
| **Spatio-temporal Multivariable final model** | PrEP use intension (%)^a^ | 3,831 | (-4,601;12,257) | **0,008 (0,002;0,014)** | 1389,10 | 0,03 |
|  | PrEP sufficient knowledge (%) | 23,146 | (-1,112;47;383) |  |  |  |
|  | Diagnosed with syphilis, gonorrhoea, or chlamydia in the previous 5 years (%) ^b^ | -22,856 | (-27,423;-13,419) |  |  |  |
|  | Always CAI with non-steady male partners (%) | -61,644 | (-74;720;-48,578) |  |  |  |
|  | Reported non-steady male CAI partners (%) | 41,254 | (33,437;49,063) |  |  |  |
|  | Having non-steady partners with diagnosed HIV (%) | 57,924 | (54,452;61,379) |  |  |  |
|  | Having steady partners living with diagnosed HIV (%) | -80,630 | (-95,460;-65,811) |  |  |  |
|  | Chemsex* in the previous 12 months (%) | 29,208 | (9,620;48,780) |  |  |  |
|  | Chemsex with multiple partners in the previous 12 months (%) | -21,107 | (-60,685;18,439) |  |  |  |
|  | Low education (%)^c^ | 23,656 | (8,188;39,113) |  |  |  |
|  | Unemployment (%) | -7,727 | (-22,380;6,914) |  |  |  |
|  | Struggling on current income (%) | -35,218 | (-54,553;-15,899) |  |  |  |

^Note: CrI = credible interval. DIC=Deviance Information Criterion, ICC= Intra-class correlation.^ **^a^** ^Defined as being quite- and very-likely to use PrEP when PrEP is available and affordable.^ **^b^** ^Data retrieved from EMIS-2017 based on non-French participants. c Defined as defined as less than three years of education beyond 16 years of age. * Chemsex, use of stimulant drugs (ecstasy/MDMA, cocaine, amphetamine, crystal methamphetamine, mephedrone or ketamine) to make sex more intense or last longer^

For the posterior prevalence and relative probability of the new PrEP uptake, we identified a similar spatial trend as what we observed from the overall PrEP uptake. Figure S11 through S13 presented the spatio-temporal distribution of the prevalence, relative probability and significance of the new PrEP uptake in France, detailed information can be found in Table S13.

We also compared the relative probability of the overall and the new PrEP uptake per region per period to identify regions with consistent lower-than-average probability of both overall and new PrEP uptake. As a result, we identified regions, namely *Auvergne-Rhône-Alpes*, *Grand Est*, *Normandi*e, *Nouvelle-Aquitaine*, *Occitanie* and *Pays de la Loire*, consistently had lower-than-average probability of both the overall and the new PrEP uptake among MSM compared to other regions from 2016S1 through 2021S1.

**Figure S11 Choropleth map of the a) Estimated prevalence of the new PrEP uptake among MSM; b) Estimated relative probability of the new PrEP uptake among MSM; c) Estimated significance of the relative probability of the new PrEP uptake compared to the overall probability in Mainland France, by Bayesian spatio-temporal ecological modelling (final model) by regions in Mainland France, 2016_S1-2021_S1**
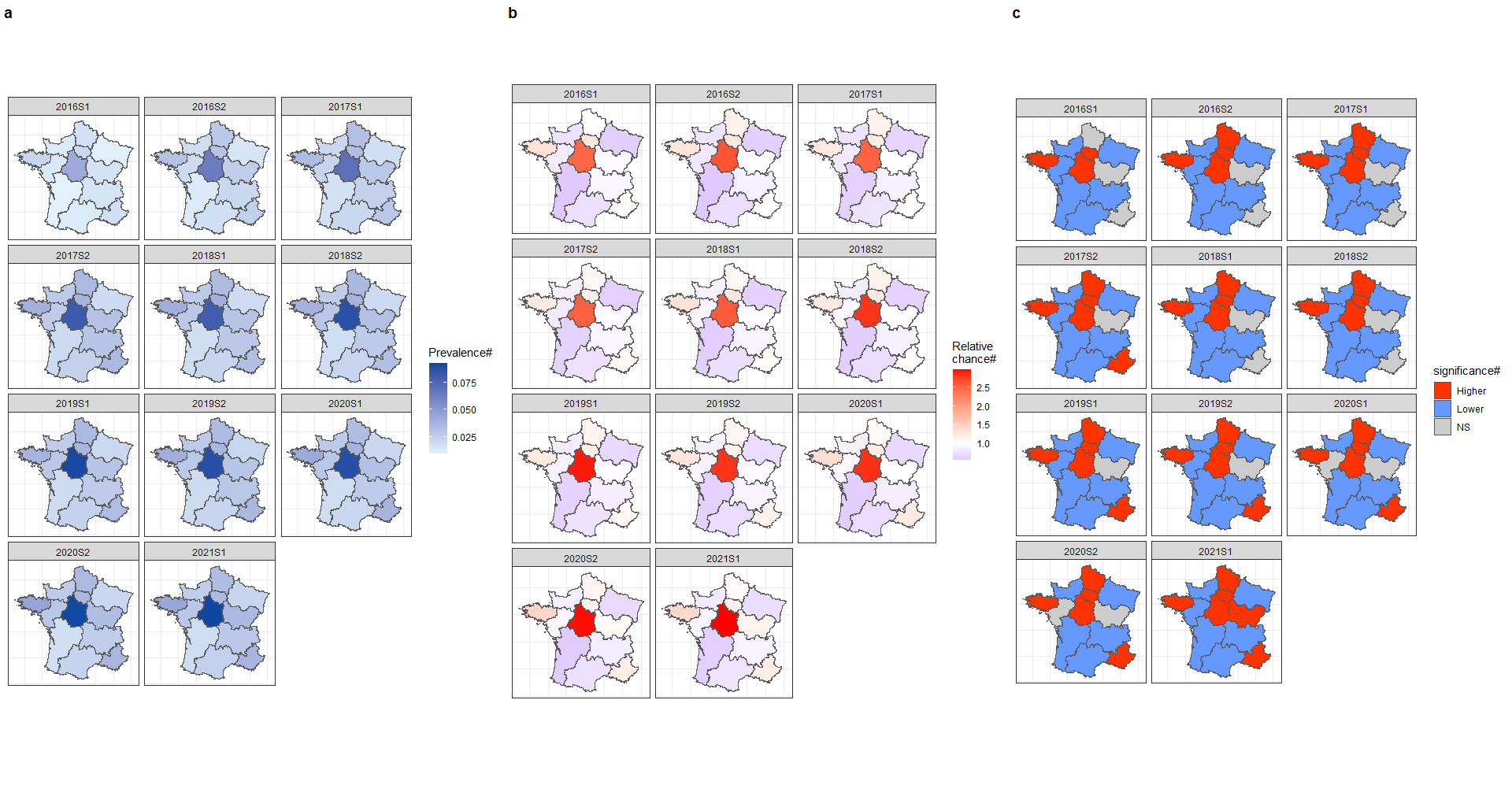


**Figure S12 Change of a) Relative probability and b) Prevalence of the new PrEP uptake among MSM by regions by Bayesian spatio-temporal ecological final model, Mainland France, 2018_S1-2020_S1**


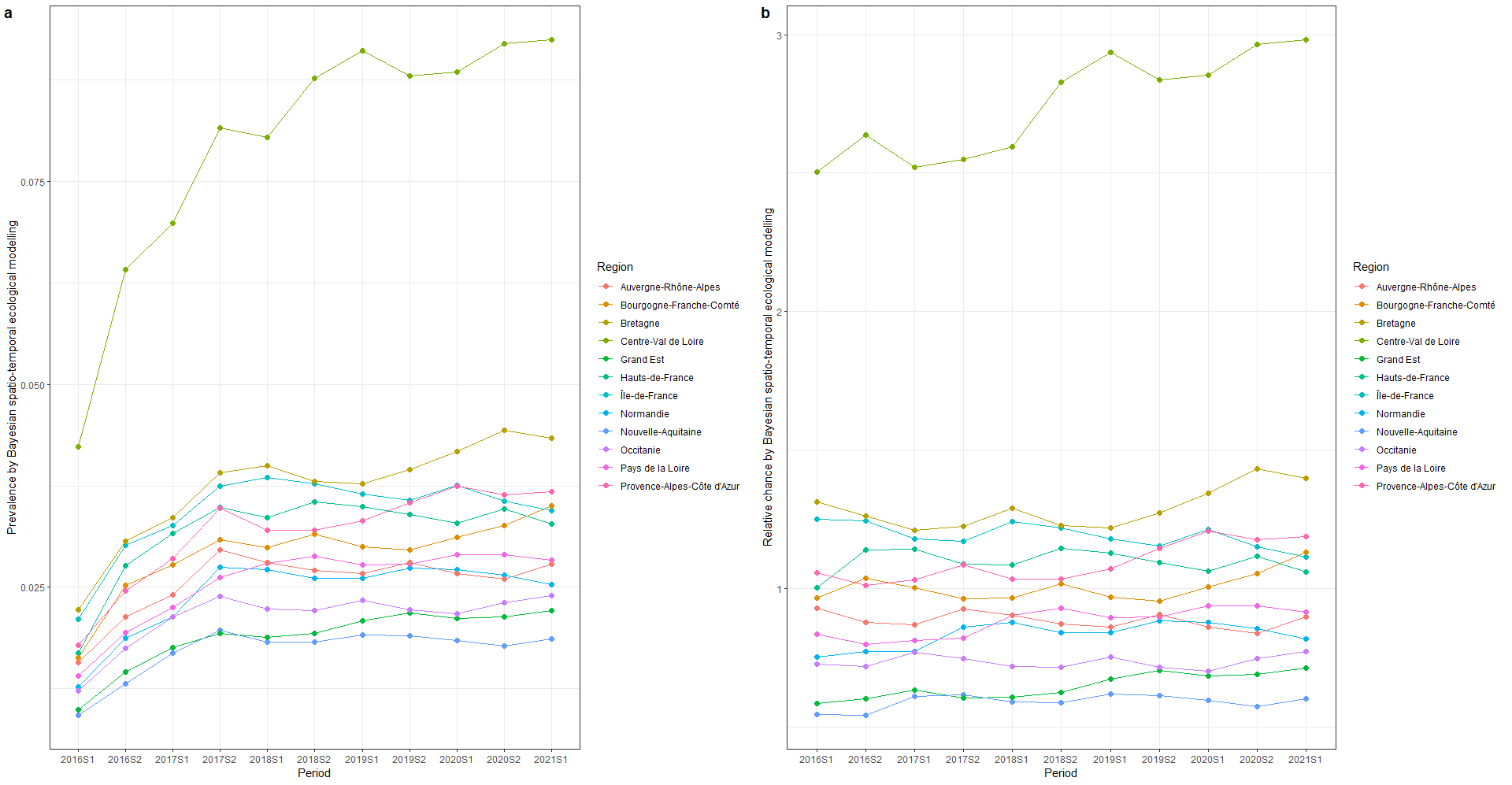


## Table S13 Prevalence and relative probability of the new PrEP uptake

| **Period** | **Region** | **Bayesian spatio-temporal**  **ecological final model** | |
| --- | --- | --- | --- |
|  |  | Relative probability  (95%CrI) | Prevalence  (95%CrI) |
| 2016  S1 | Auvergne-Rhône-Alpes | **0,929(0,873;0,99)** | 0,016(0,015;0,017) |
|  | Bourgogne-Franche-Comté | 0,967(0,88;1,057) | 0,016(0,015;0,018) |
|  | Bretagne | **1,313(1,208;1,427)** | 0,022(0,02;0,024) |
|  | Centre-Val de Loire | **2,503(2,269;2,739)** | 0,042(0,038;0,046) |
|  | Grand Est | **0,586(0,526;0,64)** | 0,01(0,009;0,011) |
|  | Hauts-de-France | 1,002(0,913;1,089) | 0,017(0,015;0,018) |
|  | Île-de-France | **1,249(1,171;1,342)** | 0,021(0,02;0,023) |
|  | Normandie | **0,753(0,675;0,831)** | 0,013(0,011;0,014) |
|  | Nouvelle-Aquitaine | **0,546(0,5;0,596)** | 0,009(0,008;0,01) |
|  | Occitanie | **0,727(0,682;0,775)** | 0,012(0,012;0,013) |
|  | Pays de la Loire | **0,835(0,759;0,912)** | 0,014(0,013;0,015) |
|  | Provence-Alpes-Côte d'Azur | 1,056(0,977;1,133) | 0,018(0,017;0,019) |
| 2016  S2 | Auvergne-Rhône-Alpes | **0,878(0,83;0,927)** | 0,021(0,02;0,023) |
|  | Auvergne-Rhône-Alpes | 1,038(0,949;1,133) | 0,025(0,023;0,028) |
|  | Bourgogne-Franche-Comté | **1,261(1,163;1,362)** | 0,031(0,028;0,033) |
|  | Bretagne | **2,638(2,412;2,871)** | 0,064(0,059;0,07) |
|  | Centre-Val de Loire | **0,601(0,553;0,649)** | 0,015(0,013;0,016) |
|  | Grand Est | **1,139(1,061;1,225)** | 0,028(0,026;0,03) |
|  | Hauts-de-France | **1,243(1,184;1,306)** | 0,03(0,029;0,032) |
|  | Île-de-France | **0,772(0,701;0,848)** | 0,019(0,017;0,021) |
|  | Normandie | **0,541(0,497;0,584)** | 0,013(0,012;0,014) |
|  | Nouvelle-Aquitaine | **0,72(0,677;0,763)** | 0,017(0,016;0,019) |
|  | Occitanie | **0,798(0,727;0,868)** | 0,019(0,018;0,021) |
|  | Pays de la Loire | 1,012(0,93;1,087) | 0,025(0,023;0,026) |
|  | Provence-Alpes-Côte d'Azur | **0,87(0,824;0,916)** | 0,024(0,023;0,025) |
| 2017  S1 | Bourgogne-Franche-Comté | 1,002(0,919;1,088) | 0,028(0,025;0,03) |
|  | Bretagne | **1,211(1,113;1,308)** | 0,034(0,031;0,036) |
|  | Centre-Val de Loire | **2,52(2,308;2,733)** | 0,07(0,064;0,076) |
|  | Grand Est | **0,633(0,587;0,682)** | 0,018(0,016;0,019) |
|  | Hauts-de-France | **1,143(1,075;1,214)** | 0,032(0,03;0,034) |
|  | Île-de-France | **1,178(1,127;1,23)** | 0,033(0,031;0,034) |
|  | Normandie | **0,772(0,702;0,843)** | 0,021(0,019;0,023) |
|  | Nouvelle-Aquitaine | **0,609(0,571;0,65)** | 0,017(0,016;0,018) |
|  | Occitanie | **0,77(0,727;0,815)** | 0,021(0,02;0,023) |
|  | Pays de la Loire | **0,813(0,75;0,879)** | 0,023(0,021;0,024) |
|  | Provence-Alpes-Côte d'Azur | 1,03(0,966;1,095) | 0,029(0,027;0,03) |
| 2017  S2 | Auvergne-Rhône-Alpes | **0,926(0,882;0,973)** | 0,03(0,028;0,031) |
|  | Bourgogne-Franche-Comté | 0,964(0,883;1,047) | 0,031(0,028;0,033) |
|  | Bretagne | **1,223(1,136;1,314)** | 0,039(0,036;0,042) |
|  | Centre-Val de Loire | **2,55(2,356;2,754)** | 0,082(0,075;0,088) |
|  | Grand Est | **0,604(0,561;0,648)** | 0,019(0,018;0,021) |
|  | Hauts-de-France | **1,088(1,026;1,152)** | 0,035(0,033;0,037) |
|  | Île-de-France | **1,171(1,123;1,219)** | 0,037(0,036;0,039) |
|  | Normandie | **0,859(0,793;0,929)** | 0,027(0,025;0,03) |
|  | Nouvelle-Aquitaine | **0,616(0,582;0,652)** | 0,02(0,019;0,021) |
|  | Occitanie | **0,747(0,709;0,785)** | 0,024(0,023;0,025) |
|  | Pays de la Loire | **0,82(0,757;0,883)** | 0,026(0,024;0,028) |
|  | Provence-Alpes-Côte d'Azur | **1,086(1,023;1,15)** | 0,035(0,033;0,037) |
| 2018  S1 | Auvergne-Rhône-Alpes | **0,903(0,863;0,944)** | 0,028(0,027;0,029) |
|  | Bourgogne-Franche-Comté | 0,966(0,889;1,046) | 0,03(0,028;0,032) |
|  | Bretagne | **1,289(1,2;1,381)** | 0,04(0,037;0,043) |
|  | Centre-Val de Loire | **2,595(2,402;2,793)** | 0,08(0,074;0,087) |
|  | Grand Est | **0,608(0,568;0,65)** | 0,019(0,018;0,02) |
|  | Hauts-de-France | **1,084(1,024;1,146)** | 0,034(0,032;0,036) |
|  | Île-de-France | **1,243(1,198;1,289)** | 0,039(0,037;0,04) |
|  | Normandie | **0,877(0,811;0,945)** | 0,027(0,025;0,029) |
|  | Nouvelle-Aquitaine | **0,589(0,557;0,622)** | 0,018(0,017;0,019) |
|  | Occitanie | **0,72(0,683;0,756)** | 0,022(0,021;0,023) |
|  | Pays de la Loire | **0,903(0,845;0,964)** | 0,028(0,026;0,03) |
|  | Provence-Alpes-Côte d'Azur | 1,035(0,977;1,092) | 0,032(0,03;0,034) |
| 2018  S2 | Auvergne-Rhône-Alpes | **0,873(0,835;0,911)** | 0,027(0,026;0,028) |
|  | Bourgogne-Franche-Comté | 1,017(0,938;1,1) | 0,032(0,029;0,034) |
|  | Bretagne | **1,227(1,145;1,312)** | 0,038(0,035;0,041) |
|  | Centre-Val de Loire | **2,829(2,637;3,029)** | 0,088(0,082;0,094) |
|  | Grand Est | **0,624(0,584;0,665)** | 0,019(0,018;0,021) |
|  | Hauts-de-France | **1,145(1,084;1,208)** | 0,035(0,034;0,037) |
|  | Île-de-France | **1,218(1,178;1,259)** | 0,038(0,037;0,039) |
|  | Normandie | **0,841(0,779;0,906)** | 0,026(0,024;0,028) |
|  | Nouvelle-Aquitaine | **0,588(0,557;0,62)** | 0,018(0,017;0,019) |
|  | Occitanie | **0,715(0,682;0,75)** | 0,022(0,021;0,023) |
|  | Pays de la Loire | **0,93(0,871;0,991)** | 0,029(0,027;0,031) |
|  | Provence-Alpes-Côte d'Azur | 1,034(0,982;1,088) | 0,032(0,03;0,034) |
| 2019  S1 | Auvergne-Rhône-Alpes | **0,861(0,825;0,897)** | 0,027(0,026;0,028) |
|  | Bourgogne-Franche-Comté | 0,968(0,893;1,045) | 0,03(0,028;0,032) |
|  | Bretagne | **1,218(1,139;1,301)** | 0,038(0,035;0,04) |
|  | Centre-Val de Loire | **2,937(2,737;3,146)** | 0,091(0,085;0,098) |
|  | Grand Est | **0,673(0,632;0,716)** | 0,021(0,02;0,022) |
|  | Hauts-de-France | **1,128(1,069;1,19)** | 0,035(0,033;0,037) |
|  | Île-de-France | **1,178(1,14;1,217)** | 0,037(0,035;0,038) |
|  | Normandie | **0,842(0,78;0,906)** | 0,026(0,024;0,028) |
|  | Nouvelle-Aquitaine | **0,618(0,586;0,652)** | 0,019(0,018;0,02) |
|  | Occitanie | **0,753(0,719;0,789)** | 0,023(0,022;0,024) |
|  | Pays de la Loire | **0,895(0,839;0,953)** | 0,028(0,026;0,03) |
|  | Provence-Alpes-Côte d'Azur | **1,07(1,016;1,126)** | 0,033(0,032;0,035) |
| 2019  S2 | Auvergne-Rhône-Alpes | **0,906(0,869;0,945)** | 0,028(0,027;0,029) |
|  | Bourgogne-Franche-Comté | 0,955(0,882;1,031) | 0,03(0,027;0,032) |
|  | Bretagne | **1,272(1,187;1,361)** | 0,039(0,037;0,042) |
|  | Centre-Val de Loire | **2,837(2,645;3,037)** | 0,088(0,082;0,094) |
|  | Grand Est | **0,705(0,66;0,751)** | 0,022(0,02;0,023) |
|  | Hauts-de-France | **1,094(1,036;1,154)** | 0,034(0,032;0,036) |
|  | Île-de-France | **1,152(1,115;1,189)** | 0,036(0,035;0,037) |
|  | Normandie | **0,882(0,816;0,95)** | 0,027(0,025;0,029) |
|  | Nouvelle-Aquitaine | **0,613(0,58;0,646)** | 0,019(0,018;0,02) |
|  | Occitanie | **0,716(0,684;0,749)** | 0,022(0,021;0,023) |
|  | Pays de la Loire | **0,898(0,842;0,956)** | 0,028(0,026;0,03) |
|  | Provence-Alpes-Côte d'Azur | **1,143(1,085;1,203)** | 0,035(0,034;0,037) |
| 2020  S1 | Auvergne-Rhône-Alpes | **0,86(0,824;0,897)** | 0,027(0,026;0,028) |
|  | Bourgogne-Franche-Comté | 1,006(0,925;1,09) | 0,031(0,029;0,034) |
|  | Bretagne | **1,345(1,25;1,444)** | 0,042(0,039;0,045) |
|  | Centre-Val de Loire | **2,854(2,658;3,06)** | 0,088(0,082;0,095) |
|  | Grand Est | **0,683(0,639;0,73)** | 0,021(0,02;0,023) |
|  | Hauts-de-France | **1,062(1,005;1,121)** | 0,033(0,031;0,035) |
|  | Île-de-France | **1,212(1,172;1,254)** | 0,038(0,036;0,039) |
|  | Normandie | **0,878(0,811;0,948)** | 0,027(0,025;0,029) |
|  | Nouvelle-Aquitaine | **0,595(0,562;0,63)** | 0,018(0,017;0,02) |
|  | Occitanie | **0,701(0,67;0,734)** | 0,022(0,021;0,023) |
|  | Pays de la Loire | 0,937(0,874;1,002) | 0,029(0,027;0,031) |
|  | Provence-Alpes-Côte d'Azur | **1,207(1,138;1,279)** | 0,037(0,035;0,04) |
| 2020  S2 | Auvergne-Rhône-Alpes | **0,838(0,803;0,873)** | 0,026(0,025;0,027) |
|  | Bourgogne-Franche-Comté | 1,052(0,964;1,145) | 0,033(0,03;0,035) |
|  | Bretagne | **1,432(1,325;1,544)** | 0,044(0,041;0,048) |
|  | Centre-Val de Loire | **2,965(2,748;3,192)** | 0,092(0,085;0,099) |
|  | Grand Est | **0,689(0,644;0,736)** | 0,021(0,02;0,023) |
|  | Hauts-de-France | **1,117(1,057;1,179)** | 0,035(0,033;0,037) |
|  | Île-de-France | **1,15(1,112;1,188)** | 0,036(0,034;0,037) |
|  | Normandie | **0,855(0,789;0,925)** | 0,027(0,024;0,029) |
|  | Nouvelle-Aquitaine | **0,573(0,541;0,605)** | 0,018(0,017;0,019) |
|  | Occitanie | **0,746(0,706;0,788)** | 0,023(0,022;0,024) |
|  | Pays de la Loire | 0,938(0,874;1,005) | 0,029(0,027;0,031) |
|  | Provence-Alpes-Côte d'Azur | **1,175(1,108;1,245)** | 0,036(0,034;0,039) |
| 2021  S1 | Auvergne-Rhône-Alpes | **0,899(0,85;0,949)** | 0,028(0,026;0,029) |
|  | Bourgogne-Franche-Comté | **1,13(1,029;1,236)** | 0,035(0,032;0,038) |
|  | Bretagne | **1,399(1,293;1,511)** | 0,043(0,04;0,047) |
|  | Centre-Val de Loire | **2,982(2,756;3,228)** | 0,092(0,085;0,1) |
|  | Grand Est | **0,713(0,662;0,767)** | 0,022(0,021;0,024) |
|  | Hauts-de-France | **1,059(1,002;1,118)** | 0,033(0,031;0,035) |
|  | Île-de-France | **1,113(1,076;1,15)** | 0,034(0,033;0,036) |
|  | Normandie | **0,816(0,753;0,882)** | 0,025(0,023;0,027) |
|  | Nouvelle-Aquitaine | **0,603(0,57;0,637)** | 0,019(0,018;0,02) |
|  | Occitanie | **0,772(0,724;0,821)** | 0,024(0,022;0,025) |
|  | Pays de la Loire | **0,915(0,85;0,986)** | 0,028(0,026;0,031) |
|  | Provence-Alpes-Côte d'Azur | **1,186(1,117;1,26)** | 0,037(0,035;0,039) |

^Note: Value with bold indicated the significant higher-(>1)/lower-(<1) than average probability of PrEP uptake among MSM in Mainland France CrI = credible interval^

# Reference

1. Weatherburn P, Hickson F, Reid DS, Marcus U, Schmidt AJ. European Men-Who-Have-Sex-With-Men Internet Survey (EMIS-2017): Design and Methods. Sexuality Research and Social Policy. 2020;17(4):543-57.

2. Sante.Publique. Nombre de découvertes d'infection à VIH selon le lieu de domicile avec un mode de contamination sexuel pour les hommes ayant des rapports sexuels avec des hommes (HSH) - tous âges 2017 [Available from: <https://geodes.santepubliquefrance.fr/#bbox=-501738,5679219,239331,257432&c=indicator&f=0&i=vih_dom.vih_mc1&s=2017&selcodgeo=06&t=a01&view=map1>.

3. Billioti de Gage S, Desplas D, Dray-Spira R. Roll-out of HIV pre-exposure prophylaxis use in France: A nationwide observational study from 2016 to 2021. The Lancet Regional Health - Europe. 2022;22:100486.

4. Wang H, Daas Cd, de Coul EO, Jonas KJ. MSM with HIV: Improving prevalence and risk estimates by a Bayesian small area estimation modelling approach for public health service areas in the Netherlands. Spatial and Spatio-temporal Epidemiology. 2023:100577.

5. Simpson D, Rue H, Riebler A, Martins TG, Sorbye SH. Penalising Model Component Complexity: A Principled, Practical Approach to Constructing Priors. Statist Sci. 2017;32(1):1-28.

6. Moraga P. Geospatial Health Data: Modeling and Visualization with R-INLA and Shiny2019.

7. Le Vu S, Le Strat Y, Barin F, Pillonel J, Cazein F, Bousquet V, et al. Population-based HIV-1 incidence in France, 2003-08: a modelling analysis. Lancet Infect Dis. 2010;10(10):682-7.

8. Ndawinz JD, Costagliola D, Supervie V. New method for estimating HIV incidence and time from infection to diagnosis using HIV surveillance data: results for France. Aids. 2011;25(15):1905-13.

9. ANSM. INFORMATIONS IMPORTANTES CONCERNANT LE BON USAGE DE L’EMTRICITABINE/TÉNOFOVIR DISOPROXIL* DANS L’INDICATION « PROPHYLAXIE PRÉ-EXPOSITION (PrEP) » AU VIH 2018 [Available from: <https://ansm.sante.fr/uploads/2021/07/05/emtricitabine-et-tenofovir-disoproxil-brochure-d-information-pds-version4-2021-juin.pdf>.

10. Annequin M, Villes V, Delabre RM, Alain T, Morel S, Michels D, et al. Are PrEP services in France reaching all those exposed to HIV who want to take PrEP? MSM respondents who are eligible but not using PrEP (EMIS 2017). AIDS Care. 2020;32(sup2):47-56.

11. Marcus U, Mirandola M, Schink SB, Gios L, Schmidt AJ. Changes in the prevalence of self-reported sexually transmitted bacterial infections from 2010 and 2017 in two large European samples of men having sex with men–is it time to re-evaluate STI-screening as a control strategy? PLOS ONE. 2021;16(3):e0248582.
